# Multicenter Validation of Clinical Sepsis Phenotypes

**DOI:** 10.1001/jamanetworkopen.2026.16134

**Published:** 2026-06-01

**Authors:** Chang Ho Yoon, Daniel Sjöholm, Kristin E. Wickstrøm, Anders Skyrud Danielsen, Christian Prebensen, John Karlsson Valik, Aleksander Rygh Holten, Erik Koldberg Amundsen, A. Sarah Walker, David W. Eyre, Valeria Vitelli, Pontus Nauclér

**Affiliations:** 1 Nuffield Department of Medicine, University of Oxford, Oxford, United Kingdom; 2 Big Data Institute, Nuffield Department of Population Health, University of Oxford, Oxford, United Kingdom; 3 Department of Medicine Solna, Clinical Epidemiology Unit, Karolinska Institutet, Karolinska University Hospital, Solna, Sweden; 4 Department of Medical Biochemistry, Blood Cell Research Group, Oslo University Hospital, Oslo, Norway; 5 Department of Medical Biochemistry, Unilabs Laboratory Medicine, Oslo, Norway; 6 Institute of Clinical Medicine, University of Oslo, Oslo, Norway; 7 Department of Infection Control and Preparedness, Norwegian Institute of Public Health, Oslo, Norway; 8 Department of Microbiology, Oslo University Hospital, Oslo, Norway; 9 Department of Infectious Diseases, Oslo University Hospital, Oslo, Norway; 10 Division of Infectious Diseases, Department of Medicine, Karolinska Institutet, Solna, Stockholm, Sweden; 11 Department of Infectious Diseases, Karolinska University Hospital, Stockholm, Sweden; 12 Department of Acute Medicine, Oslo University Hospital, Oslo, Norway; 13 Department of Life Sciences and Health, Oslo Metropolitan University, Oslo, Norway; 14 The National Institute for Health Research Health Protection Research Unit in Healthcare Associated Infections and Antimicrobial Resistance at the University of Oxford, Oxford, United Kingdom; 15 National Institute for Health Research, Oxford Biomedical Research Centre, Oxford, United Kingdom; 16 Oxford University Hospitals NHS Foundation Trust, Oxford, United Kingdom; 17 Oslo Centre for Biostatistics and Epidemiology, Department of Biostatistics, Institute of Basic Medical Sciences, University of Oslo, Oslo, Norway

## Abstract

**Importance:**

Four clinical phenotypes of sepsis based on data from electronic health records have been proposed. Although promising, the generalizability of these phenotypes remains uncertain, and multisite validation is needed.

**Objective:**

To validate, using the same methods and inclusion criteria, the 4 clinical phenotypes derived from Sepsis Endotyping in Emergency Care (SENECA) data.

**Design, Setting, and Participants:**

This multisite retrospective cohort study uses data on adult patients admitted to the emergency departments in Stockholm, Sweden (January 1, 2011, to September 1, 2023); Oxford, England (February 4, 2014, to June 20, 2021); and Oslo, Norway (January 4, 2019, to October 9, 2023) university hospitals. Included encounters are those with body fluid cultures taken, documented antibiotic administration, and Sequential Organ Failure Assessment scores of 2 or more, all within 6 hours of admission. Data analysis was conducted from November 2, 2023, to September 1, 2025.

**Main Outcomes and Measures:**

Consensus clustering with *k*-means was used to derive 4 clinical phenotypes at each site, comparing them with the SENECA-derived phenotypes, as well as with one another.

**Results:**

There were 30 865 patient encounters in Stockholm (mean [SD] age, 68 [16] years; 18 165 men [59%]), 15 575 in Oxford (mean [SD] age, 71 [18] years; 9067 men [58%]), and 1806 in Oslo (mean [SD] age, 71 [17] years; 1068 men [59%]). There was little consistency between the SENECA clinical phenotypes and each site’s own phenotypes, with a Cohen κ of 0.32 for Stockholm, 0.37 for Oslo, and 0.40 for Oxford; the Adjusted Rand Indices were 0.21 for Stockholm, 0.27 for Oslo, and 0.26 for Oxford. There was also little consistency between the phenotypes derived in Stockholm, Oxford, and Oslo.

**Conclusions and Relevance:**

This study suggests that the 4 clinical phenotypes of the SENECA data are not generalizable across 3 independent cohorts. This calls for further exploration of possible underlying sepsis subgroups using alternative approaches that mitigate the inherent stochasticity in many unsupervised and semisupervised clustering methods.

## Key Points

**Question:**

Are the 4 clinical phenotypes of sepsis derived from Sepsis Endotyping in Emergency Care (SENECA) data generalizable across independent, international patient cohorts?

**Findings:**

In this multisite retrospective cohort study of 48 246 patient encounters, there was poor consistency between the SENECA-derived phenotypes and the phenotypes derived at 3 independent clinical sites.

**Meaning:**

This study found that the 4 clinical phenotypes of sepsis derived from SENECA data lack generalizability across independent cohorts, suggesting that they may not be suitable for universal clinical application in their current form.

## Teaser

This cohort study assesses whether the 4 clinical phenotypes of sepsis derived from Sepsis Endotyping in Emergency Care data are generalizable across independent, international patient cohorts.

## Introduction

Sepsis, life-threatening organ dysfunction caused by a dysregulated host response to infection, remains a major global health challenge.^1^ Its significant heterogeneity has hindered the development of effective targeted therapies.^2,3^

An important step toward addressing this complexity was the work of Seymour et al,^4^ who identified 4 distinct sepsis phenotypes (α, β, γ, and δ) using an unsupervised machine learning clustering method applied to routinely collected electronic health record (EHR) data from emergency department (ED) patients with sepsis in the Sepsis Endotyping in Emergency Care (SENECA) study. These phenotypes, defined by readily available clinical and laboratory variables, demonstrated distinct clinical profiles, biological underpinnings, and different mortality risks. This classification raised hopes for phenotype-driven tailoring of sepsis treatments.^2^

However, the findings of the original study have yet to be externally validated outside of their site-specific contexts in a broad population of patients presenting with possible sepsis. Epidemiologic factors, prevalence of comorbidities and frailty, health care delivery practices, and availability of patient data vary across the globe. These differences could affect the manifestation and clustering of sepsis cases, raising the question of whether the SENECA phenotypes are universally applicable, with repercussions for informing therapeutic trial design and clinical guidelines. The generalizability of these phenotypes is, therefore, an open and crucial question.

This study aimed to directly address this knowledge gap by validating the SENECA sepsis phenotypes in 3 distinct health care settings outside the US. Using the same methods as the SENECA study as much as possible, including cohort selection, variables, and unsupervised clustering techniques, we assessed whether comparable sepsis phenotypes emerge in diverse populations.

## Methods

This was an international, multicenter, retrospective cohort study of observational ED cohorts in Oslo, Norway; Stockholm, Sweden; and Oxford, England. For the Oslo cohort, the study was approved by the Regional Committee for Medical and Health Research Ethics in South-East Norway. Due to the observational nature, informed patient consent was waived. Routinely measured laboratory values were exported from the laboratory databases. For the Stockholm cohort, the research was approved by the Ethical Review Authority and the Regional Ethical Review Board in Stockholm with a waiver for informed consent because of the study’s observational nature. For the Oxford cohort, deidentified data were obtained from the Infections in Oxfordshire Research Database,^5^ which has approvals from the South Central–Oxford C Research Ethics Committee, the Health Research Authority, and the national Confidentiality Advisory Group, as a research database not requiring individual patient consent. This study followed the Strengthening the Reporting of Observational Studies in Epidemiology (STROBE) reporting guideline where applicable.

### Patient Cohorts

In the Oslo cohort, adult patients (≥18 years) admitted to the ED and received by a medical response or sepsis response team between January 4, 2019, and October 9, 2023, were included in a quality register. Oslo University Hospital serves as a primary, as well as a tertiary teaching hospital, totaling 4 hospitals. For the Stockholm cohort, retrospective cohort data were collected through a research copy of Karolinska University Hospital’s EHR system, a teaching hospital with more than 1000 beds across 2 hospital sites with a tertiary care responsibility of approximately 2.4 million inhabitants. The cohort included adult patients (≥18 years) admitted to the hospital through the ED between January 1, 2011, and September 1, 2023. In Oxford, retrospective cohort data were obtained between February 4, 2014, and June 30, 2021, for adults (≥18 years) admitted to Oxford University Hospitals, a large UK teaching hospital group (4 hospitals and >1000 beds) that serves a population of approximately 650 000 with all acute care services and more than 90% of hospital services in Oxfordshire. Follow-up was censored at the earliest of 28 days or the last day the patient was known to be alive from national data or hospital records.

### Cohort Selection

Following the SENECA study, we included patients 18 years or older who met sepsis criteria within 6 hours of admission: (1) suspected infection indicated by 1 or more culture specimens (urine, blood, cerebrospinal fluid) with documented administration of systemic antibiotics and (2) signs of organ dysfunction, defined as a Sequential Organ Failure Assessment score of 2 or more.^4,6^

### Statistical Analysis

Data analysis was conducted from November 2, 2023, to September 1, 2025. We used the 29 variables identified by Seymour et al^4^: demographic variables, vital signs, markers of inflammation, and organ dysfunction (eTable 1 in Supplement 1). Where exact variables were unavailable, we used the closest equivalents: neutrophil granulocytes (as a percentage of total leukocytes) instead of bands and urea instead of blood urea nitrogen (eTable 1 in Supplement 1). These were aliased to match what was used by Seymour et al.^4^ The Oxford and Stockholm datasets included all 29 variables, and the Oslo dataset had 26 variables (missing aspartate aminotransferase, bicarbonate, and the Elixhauser comorbidity score; eTable 1 in Supplement 1).

As in the study by Seymour et al,^4^ at each site, we independently standardized the data and transformed nonnormal data; nonnormal data were log transformed and standardized to a mean (SD) of 0 (1) (eTable 1 in Supplement 1). Multiple imputation with chained equations with predictive mean matching was used in R, version 4.5 (R Project for Statistical Computing), for handling missing data, and 11 imputed datasets were created,^4^ with the convergence plots indicating that the imputation process had converged. Consensus clustering was performed on each of the 11 imputed datasets with *k*-means, prespecifying *k* = 4.^4,7,8^ We explored only 4 clusters, as the main goal was to replicate the method used to derive the SENECA phenotypes. Each dataset in the consensus clustering run was resampled 20 times with a resampling number of 0.8. Results were analyzed on a pooled matrix from the run of consensus clustering by taking the mean of the individual consensus clustering matrices, combined with a median dataset of the 11 imputed datasets. The output for each site was a set of clusters, identified by their midpoint in the scaled variable space (ie, their centroid). This centroid defined the phenotype. We performed the clustering at each site to derive 4 site-specific clinical phenotypes, first based on 26 variables (Oslo, Oxford, and Stockholm), and then with all 29 variables (Oxford and Stockholm). The list of centroid positions was shared between all 3 sites and applied on each dataset in order. Finally, we compared the SENECA-derived phenotypes with the clinical phenotypes derived at each site. We compared phenotype assignment in a pairwise manner (individual site-specific phenotypes with 26 variables for SENECA vs Oslo, Oxford, and Stockholm; 29 variables for SENECA vs Oxford and Stockholm). To facilitate comparison, we relabeled all phenotypes 1 to 4 from each cohort to be as close as possible in the scaled space to the α to δ phenotypes from SENECA (ie, 1 to α, 2 to β, 3 to γ, 4 to δ).

To quantify phenotypic concordance, we used Cohen κ for each SENECA-to-specific-site comparison (interrater agreement value of 0-1 between 2 sites), and Fleiss κ for comparison across all sites (interrater agreement between 3 sites).^9,10^ In addition, to quantify phenotypic concordance while accounting for the arbitrary nature of phenotypic labeling, we also calculated the Adjusted Rand Index.^11,12^ Through alluvial plots, we visually assessed the reproducibility of phenotypes. Finally, to determine which variables were most associated with phenotype assignment, we calculated the Pearson correlation coefficients or, more specifically, the special case of point biserial correlation coefficients. Here, patients were labeled as 1 if in a specific phenotype and as 0 otherwise, with correlation coefficients calculated for each of the 26 or 29 input variables. We performed analyses using R, version 4.5 (R Project for Statistical Computing).^13^

## Results

### Cohort Descriptions

The cohorts (Stockholm: n = 30 865, mean [SD] age, 68 [16] years; 18 165 men [59%] and 12 700 women [41%]; Oxford: n = 15 575, mean [SD] age, 71 [18] years; 9067 men [58%] and 6508 women [42%]; and Oslo: n = 1806, mean [SD] age, 71 [17] years; 1068 men [59%] and 738 women [41%]) were broadly similar across the 26 to 29 variables (Table; eFigures 1, 3, and 4 in Supplement 1). The Stockholm cohort of 30 865 patients comprised 18 165 (59%) males, similar to Oxford (9067 of 15575 [58%]) and Oslo (1068 of 1806 [59%]), while SENECA’s derivation cohort had 50% males (10 022 of 20 189).^4^ The mean (SD) Elixhauser comorbidity index, where available, was lower in SENECA’s derivation cohort (Stockholm, 2.6 [2.0]; Oxford, 2.8 [2.0]; SENECA, 1.8 [1.2]) (Table). The pulmonary variables of respiratory rate and oxygen saturation were similar across all cohorts; the mean (SD) partial pressure of oxygen was similar in Stockholm (77 [40] mm Hg), Oxford (71 [31] mm Hg), and Oslo (75 [40] mm Hg) (Table), while SENECA’s derivation cohort had higher values (123 [89] mm Hg) (to convert to kilopascals, multiply by 0.133). Cardiovascular variables (bicarbonate, heart rate, lactate, systolic blood pressure, and troponin) were similar across all cohorts (eg, the median systolic blood pressure was 111 mm Hg [IQR, 97-129 mm Hg] in Stockholm; 113 mm Hg [IQR, 98-132 mm Hg] in Oxford; and 118 mm Hg [IQR, 96-140 mm Hg] in Oslo; and 110 mm Hg [IQR, 93-128 mm Hg] in SENECA). Kidney and hepatic variables (eg, creatinine, sodium, alanine aminotransferase, aspartate aminotransferase, and bilirubin), with the exception of blood urea nitrogen, were also similar across cohorts (eg, the median creatinine level was 1.2 mg/dL [IQR, 0.8-1.7 mg/dL] in Stockholm; 1.4 mg/dL [IQR, 0.9-2.2 mg/dL] in Oxford; 1.1 mg/dL [IQR, 0.8-1.7 mg/dL] in Oslo; and 1.4 mg/dL [IQR, 1.0-2.2 mg/dL] in SENECA [to convert to micromoles per liter, multiply by 88.4]). Hematologic variables (hemoglobin, platelets, and international normalized ratio) were broadly similar across all cohorts (eg, the mean [SD] hemoglobin level was 11 [2.3] g/dL in Stockholm; 11 [2.5] g/dL in Oxford; 13 [2.3] g/dL in Oslo; and 12 [2] g/dL in SENECA [to convert to grams per liter, multiply by 10.0]). The neurologic variable Glasgow Coma Scale score was lower in SENECA (mean [SD], 14 [1.8] in Stockholm; 14 [2.5] in Oxford; 14 [2.6] in Oslo; and 11.4 [4] in SENECA). For variables of inflammation (erythrocyte sedimentation rate, C-reactive protein, leucocytes, bands, and temperature), the 4 cohorts were similar except for bands, where values were lower in SENECA (explained in part by bands being replaced by neutrophils in other cohorts).

**Table.** Summary Statistics by Site^a^

Variable	Stockholm (n = 30 865)	Oxford (n = 15 575)	Oslo (n = 1806)
Age, mean (SD), y	68 (16)	71 (18)	71 (17)
Sex, No. (%)			
Male	18 165 (59)	9067 (58)	1068 (59)
Female	12 700 (41)	6508 (42)	738 (41)
Alanine aminotransferase, median (IQR), IU/L	26 (16-50)	26 (16-52)	22 (14-38)
Albumin, mean (SD), g/dL	2.9 (0.6)	3.2 (0.6)	3.7 (0.6)
Bilirubin, median (IQR), mg/dL	0.7 (0.4-1.2)	1.1 (0.6-1.8)	0.6 (0.4-1.0)
Blood urea nitrogen, median (IQR), mg/dL^b^	36 (21-60)	29 (18-49)	23 (16-35)
Chloride, mean (SD), mEq/L	104 (6.6)	105 (7.3)	99 (7.1)
Creatinine, median (IQR), mg/dL	1.2 (0.8-1.7)	1.4 (0.9-2.2)	1.1 (0.8-1.7)
C-reactive protein, median (IQR), mg/L	105 (44-201)	126 (53-219)	82 (30-180)
Erythrocyte sedimentation rate, median (IQR), mm/h	62 (32-91)	55 (26-92)	41 (20-72)
Glasgow Coma Scale score, mean (SD)	14 (1.8)	14 (2.5)	14 (2.6)
Hemoglobin, mean (SD), g/dL	11 (2.3)	11 (2.5)	13 (2.3)
Heart rate, mean (SD), beats/min	106 (22)	97 (21)	107 (23)
International normalized ratio, median (IQR)	1.2 (1.0-1.3)	1.1 (1.1-1.3)	1.1 (1.0-1.3)
Lactate, median (IQR), mg/dL	16 (11-25)	18 (12-28)	15 (9.9-25)
Leukocytes, median (IQR), ×10^3^/μL	12 (6.9-16)	14 (9.4-19)	12 (8.7-17)
Pao_2_, mean (SD), mm Hg	77 (40)	71 (31)	75 (40)
Respiratory rate, mean (SD), breaths/min	26 (8.0)	21 (5.5)	29 (11)
Spo_2_, median (IQR), %	93 (89-95)	95 (94-97)	93 (90-96)
Systolic blood pressure, median (IQR), mm Hg	111 (97-129)	113 (98-132)	118 (96-140)
Sodium, mean (SD), mEq/L	137 (5.8)	138 (6.0)	138 (5.8)
Temperature, mean (SD), °C	38.2 (1.1)	37.1 (1.1)	37.7 (1.5)
Thrombocytes, median (IQR), ×10^9^/L	205 (131-285)	186 (128-252)	229 (171-296)
Troponin, median (IQR), ng/mL	0.038 (0.020-0.077)	0.060 (0.022-0.21)	0.040 (0.020-0.084)
Bands, median (IQR), %^c^	81 (67-89)	85 (78-90)	86 (78-90)
Aspartate aminotransferase, median (IQR), IU/L	31 (21-56)	49 (24-134)	NA
Bicarbonate, mean (SD), mEq/L	24 (4.2)	22 (4.6)	NA
Elixhauser comorbidity index, mean (SD)	2.6 (2.0)	2.8 (2.0)	NA

Abbreviations: NA, not applicable; Pao_2_, partial pressure of oxygen; Spo_2_, oxygen saturation.

SI conversion factors: To convert alanine aminotransferase to microkatals per liter, multiply by 0.0167; albumin to grams per liter, multiply by 10.0; bilirubin to micromoles per liter, multiply by 17.104; blood urea nitrogen to millimoles per liter, multiply by 0.357; chloride to millimoles per liter, multiply by 1.0; creatinine to micromoles per liter, multiply by 88.4; C-reactive protein to milligrams per liter, multiply by 10.0; hemoglobin to grams per liter, multiply by 10.0; lactate to millimoles per liter, multiply by 0.111; Pao_2_ to kilopascals, multiply by 0.133; sodium to millimoles per liter, multiply by 1.0; troponin to micrograms per liter, multiply by 1.0; aspartate aminotransferase to microkatals per liter, multiply by 0.0167; and bicarbonate to millimoles per liter, multiply by 1.0.

^a^ Patient demographic, clinical, hematologic, and biochemical variables used for phenotyping by site. There were 29 variables in total, with 26 common across the 3 sites. The additional 3 variables in the Oxford and Stockholm cohorts are found separately at the bottom of the table.

^b^ BUN = urea used as a proxy.

^c^ Bands = neutrophils used as a proxy.

The proportions of missing values in all 3 cohorts were generally lower than in the SENECA dataset (eFigure 2 in Supplement 1) with respect to the 29 variables used by Seymour et al^4^ (eTables 1 and 2 in Supplement 1).^14^ The Oslo cohort had the most complete data (eFigure 2 in Supplement 1), with only chloride, troponin, aspartate aminotransferase, bicarbonate, and the Elixhauser comorbidity index exhibiting more than 20% of data missing. Meanwhile, the Stockholm cohort had more than 20% of data missing for 13 of the 29 variables, the Oxford cohort had more than 20% of data missing for 8 of the 29, and the SENECA cohort had more than 20% of data missing for 21 of 29 variables (12 of which had >50% of data missing).

### Differences in Phenotype Assignment

**Figure 1.**
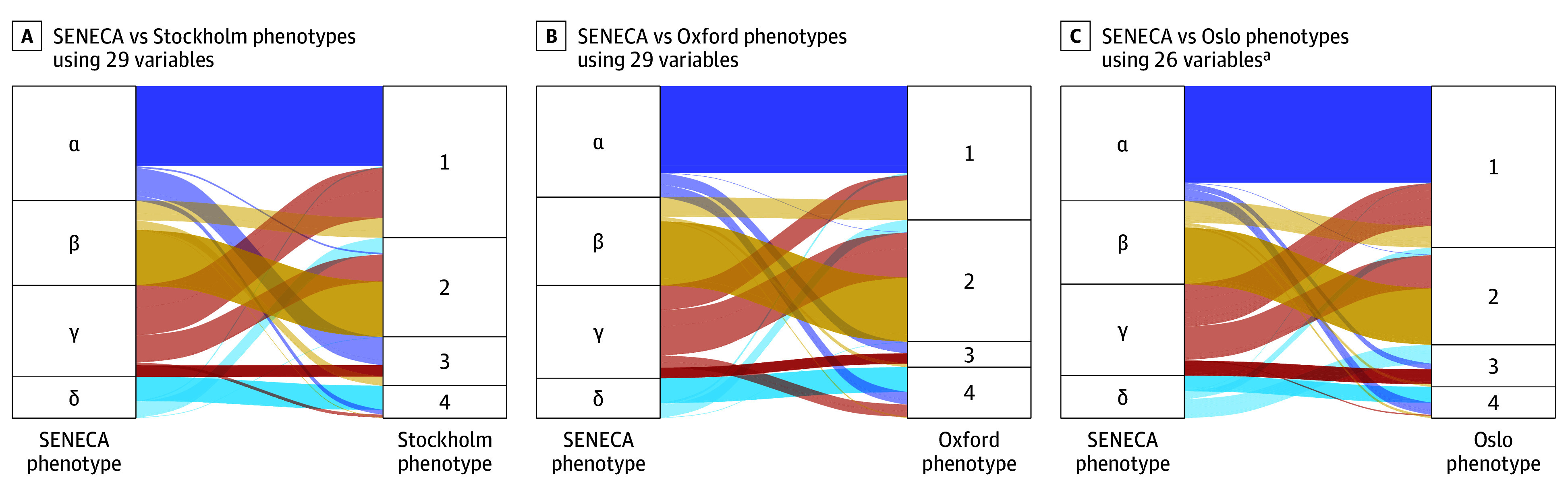
Alluvial Plots Showing Differences in Phenotype Assignment When Comparing SENECA (Sepsis Endotyping in Emergency Care) Phenotype Assignments With Site-Specific Phenotypes Patients at each site were assigned to clusters using the shortest Euclidean distance to SENECA centroids. The colored bands show the differences in cluster assignment (eg, between α using SENECA centroids and 1 for other site-specific centroids). Colors are based on SENECA phenotypes with phenotype ordering to match as close as possible among sites. Cohen κ coefficients for each site compared with SENECA are 0.32 for Stockholm, 0.40 for Oxford, and 0.37 for Oslo. The corresponding Adjusted Rand Indices compared with SENECA are 0.21 for Stockholm, 0.26 for Oxford, and 0.27 for Oslo. ^a^Three variables were unavailable.

First, we compared SENECA-derived phenotype assignments with phenotype assignments based on consensus clustering performed at each site (Figure 1). Whether using all 29 variables in the original SENECA-derived phenotypes (Stockholm, Figure 1A; Oxford, Figure 1B) or 26 variables in Oslo (Figure 1C), we observed a low level of overlap in these pairwise comparisons, with a Cohen κ of 0.32 for Stockholm, 0.37 for Oslo, and 0.40 for Oxford. The Adjusted Rand Indices were correspondingly low at 0.21 for Stockholm, 0.27 for Oslo, and 0.26 for Oxford.

**Figure 2.**
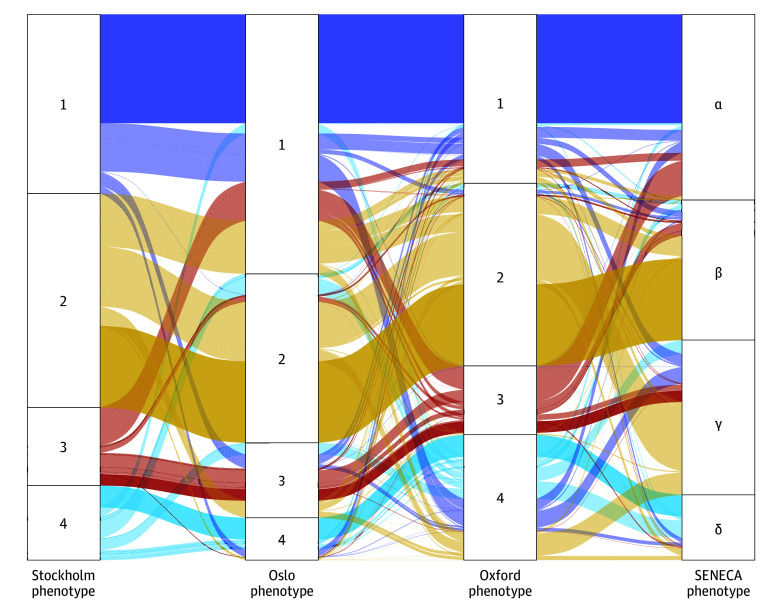
Alluvial Plot Showing Differences in Phenotype Assignment Based on Phenotypes Derived From Individual Sites Including From SENECA (Sepsis Endotyping in Emergency Care) In this example, patients are from the Stockholm dataset with the common set of 26 variables. The left-most column is the cluster assignment from the Stockholm clustering result (ie, calculated using the same dataset; meanwhile, “Oslo” indicates the assignment of Stockholm patients to the centroids derived from the Oslo dataset, based on the shortest Euclidean distance for each Stockholm patient; similarly for “Oxford” and “SENECA” with their respective site-derived centroids. Coloring is according to Stockholm phenotypes, with phenotype ordering as close as possible to SENECA’s. Fleiss κ for agreement is 0.47, and the percentage agreement among all is 41%. Similar figures for the Oxford and Oslo datasets are in Supplement 1. The Adjusted Rand Indices compared with Stockholm are 0.32 for Oslo, 0.57 for Oxford, and 0.30 for SENECA.

Second, we mapped how phenotype assignments varied when applying site-specific phenotypes to the patients of any given site (using the 26 common variables); for example, for Stockholm patients (Figure 2), while there was partial concordance with a Fleiss κ of 0.47 (Adjusted Rand Index of 0.32 for Oslo, 0.57 for Oxford, and 0.30 for SENECA), there was also substantial inconsistency in how they were classified using clustering models from other sites (eFigures 5-8 in Supplement 1).

### Phenotype Descriptions

To understand the possible reasons for the differences in phenotype assignment, first we explored phenotype characteristics; second, we identified the characteristics most correlated with phenotype assignment. The original SENECA phenotypes were described as follows: α phenotype comprised the youngest phenotypic age and had less organ dysfunction (reflected by fewer abnormal laboratory test values); β phenotype was older and had more comorbidities and more severe kidney dysfunction; γ phenotype had more pronounced inflammation variables, higher temperatures, and lower albumin levels; and δ phenotype had more hypotension, higher lactate levels, and deranged aminotransferase levels.^4^ The 28-day in-hospital mortality rates were 2% for α phenotype, 5% for β phenotype, 15% for γ phenotype, and 32% for δ phenotype.

Using the 26 variables available in the Oslo cohort to derive its own phenotypes (eTable 3 in Supplement 1), Oslo phenotype 1 was older (than other Oslo phenotypes) with no standout variables differentiating it from the rest; Oslo phenotype 2 was older, with more severe kidney dysfunction and inflammation; Oslo phenotype 3 was younger, with greater hepatic dysfunction and higher international normalized ratios; and Oslo phenotype 4 was younger with more neurologic deficits (lower Glasgow Coma Scale score). These characteristics were corroborated by the variables most correlated with phenotype assignment (eTable 6 in Supplement 1). Applying SENECA’s phenotypes to Oslo’s cohort, older patients with more severe kidney dysfunction tended toward both β and δ phenotypes; the original γ phenotype characteristics distributed across β, γ, and δ phenotypes; and δ phenotype could be described similarly to the original (albeit with more severe kidney dysfunction and lower albumin levels relative to other phenotypes). The 28-day in-hospital mortality rates of these SENECA phenotypes applied to Oslo data were 10% for Oslo phenotype 1, 19% for Oslo phenotype 2, 18% for Oslo phenotype 3, and 44% for Oslo phenotype 4.

Using all 29 variables to derive Oxford’s own phenotypes (eTable 4 in Supplement 1), Oxford phenotype 1 was older but had fewer comorbidities with no standout variables; Oxford phenotype 2 was older and had more comorbidities, with more kidney dysfunction and inflammation; Oxford phenotype 3 was younger and had more cytopenia (lower bands, hemoglobin, leucocytes, thrombocytes); and Oxford phenotype 4 had greater hepatic dysfunction and higher lactate levels. These characteristics were corroborated by the variables most correlated with phenotype assignment (eTable 7 in Supplement 1). Applying SENECA’s phenotypes to Oxford’s cohort, older patients with more severe kidney dysfunction tended toward both β and δ; the original γ phenotype characteristics appeared distributed across β, γ, and δ; and δ phenotype also comprised higher lactate and aminotransferase levels, although hypotension appeared more characteristic of γ and δ. The 28-day in-hospital mortality rates of these SENECA phenotypes applied to Oxford data were 7% for Oxford phenotype 1, 14% for Oxford phenotype 2, 12% for Oxford phenotype 3, and 21% for Oxford phenotype 4.

Again, using all 29 variables to derive Stockholm’s own phenotypes (eTable 5 in Supplement 1), Stockholm phenotype 1 had fewer comorbidities and fewer abnormal variables; Stockholm phenotype 2 was older and had more comorbidities with more severe kidney dysfunction and inflammation; Stockholm phenotype 3 was younger and had more cytopenia (bands, hemoglobin, leucocytes, and thrombocytes); and Stockholm phenotype 4 had greater hepatic dysfunction and higher lactate levels. These characteristics were corroborated by the variables most correlated with phenotype assignment (eTable 8 in Supplement 1). Applying SENECA’s phenotypes to Stockholm’s cohort, older patients with more severe kidney dysfunction tended toward both β and δ; the original γ features of greater inflammation, lower albumin levels, and higher temperatures were characteristic of β, γ, and δ; and δ phenotype also comprised higher lactate and aminotransferase levels, although hypotension characterized both γ and δ. The 28-day in-hospital mortality rates of these SENECA phenotypes applied to Stockholm data were 5% for Stockholm phenotype 1, 13% for Stockholm phenotype 2, 19% for Stockholm phenotype 3, and 33% for Stockholm phenotype 4.

## Discussion

We present the results of a large multicenter validation study of the 4 clinical sepsis phenotypes derived in the SENECA study.^4^ Using near-identical methods with a variable set as close to the original as possible, we could only partially replicate the SENECA-derived phenotypes. Phenotypes with differential mortality rates were identified, as would be expected using multidimensional data-partitioning or clustering approaches; however, by the use of cluster similarity metrics and inspection of alluvial plots of differential phenotype assignment, we were not able to closely reproduce the SENECA-derived phenotypes. Moreover, even with broadly similar variable distributions across the Stockholm, Oxford, and Oslo datasets, no clear patterns emerged when mapping the 4 phenotypes between sites.

Our results are consistent with 2 recent studies that found sepsis phenotypes derived in the ED setting to lack reproducibility, not only between sites but also within a single site, in part due to patients presenting at different stages of sepsis (particularly in the hyperacute setting)^15,16^; however, to our knowledge, this is the first direct validation study, including attempted methodological reproduction, of the SENECA-derived sepsis phenotypes. Our findings run counter to the external validation originally performed by Seymour et al^4^ on cohorts of patients with severe sepsis (in clinical trials) and pneumonia, cohorts arguably more dissimilar to the SENECA phenotype derivation patients than our studies’ ED populations with possible sepsis. This seemingly paradoxical outcome lends support to the importance of performing validation in multiple different cohorts.

In this instance, we repeated an unsupervised clustering approach, for which models from one site cannot be transferred to another (ie, the method and its results [in the form of cluster centroids] were transferred rather than the model per se). Nongeneralizability may reflect cluster instability (despite attempts to mitigate it by consensus clustering) or the need to consider nontraditional biomarkers for clearer phenotypic separation.^17,18^ It is also possible that 4 clusters is not the optimal cluster number since it might fail to capture the granularity and inherent heterogeneity of the syndrome.

### Limitations

This study has some limitations. By enumerating both the specific limitations of this study and those of widely applied validation standards of clinical machine learning models, we argue that the minutiae of the data modeling itself and the nature of validation matter. First, clustering methods such as *k*-means are inherently stochastic with no consensus on how to maximize their generalizability (possibly hindered further by an assumption of cluster convexity that might be unrealistic in a clinical setting). Second, multiple imputation, even when accounting for nonlinear interactions, is not a panacea^19^; the original SENECA phenotypes were based on imputed variables, seemingly with 1 of 11 imputed datasets being selected for analyses.^4^ The high level of missingness in our study and the SENECA study is a limitation. The exclusion of highly missing variables, especially when missingness may not be at random, may result in less-biased and more reproducible results. Third, both the SENECA study and our study considered only static snapshots of sepsis; in different health care systems and cultures, patients with potential sepsis may present at different phases of the illness, along with differential waiting times before their physiological status in the ED is captured. Therefore, instead of a single time window, time-varying variables may help address this problem while introducing more complexity (eg, data sparsity and temporal phase shifts of the same underlying sepsis phenotype at the point of data capture).

## Conclusions

This study suggests that the 4 clinical phenotypes of the SENECA data are not generalizable across 3 independent cohorts. Despite the promise of sepsis phenotyping, a revolution in diagnostics or therapeutics is yet to come to fruition. Some have therefore argued for a more longitudinal and/or multimodal (eg, with omics) approach, while others are seeking to incorporate treatment responses.^20^ However, many of these potential solutions will only render the validation problem more intractable by exponentially magnifying the curse of dimensionality. Rather than dismissing sepsis phenotyping based only on routine clinical data, we would argue in the first instance for a prudent exploration of possible underlying subgroups with a more principled, statistical approach to validation that accounts for inherent stochasticity in unsupervised and semisupervised phenotyping methods. One such approach could be more advanced functional clustering methods capable of handling time-dependent variables, hence capturing the dynamics of sepsis.

## Notes

Supplement 1. **eFigure 1. **Flowchart of Patient Selection in the Oxford, Oslo and Stockholm Cohorts (Left: Oxford, Middle: Oslo, Right: Stockholm) **eFigure 2. **Proportions of Missingness by Variable in the Oslo, Oxford and Stockholm Cohorts **eFigure 3.** Boxplots of Scaled Medians and IQR by Variable and Cohort **eFigure 4. **Boxplots of Raw Medians and IQR by Variable and Cohort **eFigure 5. **Alluvial Plot Showing Differences in Patients From a Given Site Being Assigned to Phenotypes Derived From Individual Sites Incl. SENECA **eFigure 6.** Alluvial Plot Showing Differences in Patients From a Given Site Being Assigned to Phenotypes Derived From Individual Sites Incl. SENECA **eFigure 7. **Alluvial Plot Showing Differences in Patients From a Given Site Being Assigned to Phenotypes Derived From Individual Sites Incl. SENECA Using All 29 Variables **eFigure 8.** Alluvial Plot Showing Differences in Patients From a Given Site Being Assigned to Phenotypes Derived From Individual Sites Incl. SENECA Using All 29 Variables **eTable 1.** Variables and Their Units Included in the Analysis, as Originally Selected in the SENECA Data Analyzed by Seymour et al [1], and Using the Same Transformations **eTable 2. **Variables and Their Units in the SENECA, Stockholm, Oslo and Oxford Datasets **eTable 3. **Characteristics of the Four Phenotypes Derived From Applying the Same Consensus Clustering Approach on the Oslo Cohort (1os, 2os, 3os, 4os), and the Characteristics of the Four Phenotypes When Oslo Patients Are Assigned to the Closest SENECA Phenotypes in Euclidean Space **eTable 4. **Characteristics of the Four Phenotypes Derived From Applying the Same Consensus Clustering Approach on the Oxford Cohort (1ox, 2ox, 3ox, 4ox), and the Characteristics of the Four Phenotypes When Oxford Patients Are Assigned to the Closest SENECA Phenotypes in Euclidean Space **eTable 5. **Characteristics of the Four Phenotypes Derived From Applying the Same Consensus Clustering Approach on the Stockholm Cohort (1st, 2st, 3st, 4st), and the Characteristics of the Four Phenotypes When Stockholm Patients Are Assigned to the Closest SENECA Phenotypes in Euclidean Space **eTable 6. **Highlight Table of Pearson Correlation Coefficients Between Scaled Variable Values and Phenotype Assignment of the Oslo Cohort Using Phenotypes Derived From Each Site Where 26 Variables Were Available (Oslo, Oxford, Stockholm, SENECA) **eTable 7. **Highlight Table of Pearson Correlation Coefficients Between Scaled Variable Values and Phenotype Assignment of the Oxford Cohort Using Phenotypes Derived From Sites Where 29 Variables Were Available (Oxford, Stockholm, SENECA) **eTable 8. **Highlight Table of Pearson Correlation Coefficients Between Scaled Variable Values and Phenotype Assignment of the Stockholm Cohort Using Phenotypes Derived From Sites Where 29 Variables Were Available (Oxford, Stockholm, SENECA)
eReference.

Supplement 2.
Data Sharing Statement
