# Supplementary material for: Multicenter Validation of Clinical Sepsis Phenotypes
Source: JAMA Netw Open. 2026 Jun 1;9(6):e2616134. doi: 10.1001/jamanetworkopen.2026.16134 (PMC13227316; doi:10.1001/jamanetworkopen.2026.16134)
Supplement: Supplement 1. — eFigure 1. Flowchart of Patient Selection in the Oxford, Oslo and Stockholm Cohorts (Left: Oxford, Middle: Oslo, Right: Stockholm) eFigure 2. Proportions of Missingness by Variable in the Oslo, Oxford and Stockholm Cohorts eFigure 3. Boxplots of Scaled Medians and IQR by Variable and Cohort eFigure 4. Boxplots of Raw Medians and IQR by Variable and Cohort eFigure 5. Alluvial Plot Showing Differences in Patients From a Given Site Being Assigned to Phenotypes Derived From Individual Sites Incl. SENECA eFigure 6. Alluvial Plot Showing Differences in Patients From a Given Site Being Assigned to Phenotypes Derived From Individual Sites Incl. SENECA eFigure 7. Alluvial Plot Showing Differences in Patients From a Given Site Being Assigned to Phenotypes Derived From Individual Sites Incl. SENECA Using All 29 Variables eFigure 8. Alluvial Plot Showing Differences in Patients From a Given Site Being Assigned to Phenotypes Derived From Individual Sites Incl. SENECA Using All 29 Variables eTable 1. Variables and Their Units Included in the Analysis, as Originally Selected in the SENECA Data Analyzed by Seymour et al [1], and Using the Same Transformations eTable 2. Variables and Their Units in the SENECA, Stockholm, Oslo and Oxford Datasets eTable 3. Characteristics of the Four Phenotypes Derived From Applying the Same Consensus Clustering Approach on the Oslo Cohort (1os, 2os, 3os, 4os), and the Characteristics of the Four Phenotypes When Oslo Patients Are Assigned to the Closest SENECA Phenotypes in Euclidean Space eTable 4. Characteristics of the Four Phenotypes Derived From Applying the Same Consensus Clustering Approach on the Oxford Cohort (1ox, 2ox, 3ox, 4ox), and the Characteristics of the Four Phenotypes When Oxford Patients Are Assigned to the Closest SENECA Phenotypes in Euclidean Space eTable 5. Characteristics of the Four Phenotypes Derived From Applying the Same Consensus Clustering Approach on the Stockholm Cohort (1st, 2st, 3st, 4st), and the Characteristics of [file jamanetwopen-e2616134-s001.pdf]

# Supplementary Online Content

Yoon CH, Sjöholm D, Wickstrøm KE, et al. Multicenter validation of clinical sepsis phenotypes. *JAMA Netw Open.* 2026;9(6):e2616134.

doi:10.1001/jamanetworkopen.2026.16134

**eFigure 1.** Flowchart of Patient Selection in the Oxford, Oslo and Stockholm Cohorts (Left: Oxford, Middle: Oslo, Right: Stockholm)
**eFigure 2.** Proportions of Missingness by Variable in the Oslo, Oxford and Stockholm Cohorts
**eFigure 3.** Boxplots of Scaled Medians and IQR by Variable and Cohort
**eFigure 4.** Boxplots of Raw Medians and IQR by Variable and Cohort
**eFigure 5.** Alluvial Plot Showing Differences in Patients From a Given Site Being Assigned to Phenotypes Derived From Individual Sites Incl. SENECA
**eFigure 6.** Alluvial Plot Showing Differences in Patients From a Given Site Being Assigned to Phenotypes Derived From Individual Sites Incl. SENECA
**eFigure 7.** Alluvial Plot Showing Differences in Patients From a Given Site Being Assigned to Phenotypes Derived From Individual Sites Incl. SENECA Using All 29 Variables
**eFigure 8.** Alluvial Plot Showing Differences in Patients From a Given Site Being Assigned to Phenotypes Derived From Individual Sites Incl. SENECA Using All 29 Variables
**eTable 1.** Variables and Their Units Included in the Analysis, as Originally Selected in the SENECA Data Analyzed by Seymour et al [1], and Using the Same Transformations
**eTable 2.** Variables and Their Units in the SENECA, Stockholm, Oslo and Oxford Datasets
**eTable 3.** Characteristics of the Four Phenotypes Derived From Applying the Same Consensus Clustering Approach on the Oslo Cohort (1os, 2os, 3os, 4os), and the Characteristics of the Four Phenotypes When Oslo Patients Are Assigned to the Closest SENECA Phenotypes in Euclidean Space
**eTable 4.** Characteristics of the Four Phenotypes Derived From Applying the Same Consensus Clustering Approach on the Oxford Cohort (1ox, 2ox, 3ox, 4ox), and the Characteristics of the Four Phenotypes When Oxford Patients Are Assigned to the Closest SENECA Phenotypes in Euclidean Space
**eTable 5.** Characteristics of the Four Phenotypes Derived From Applying the Same Consensus Clustering Approach on the Stockholm Cohort (1st, 2st, 3st, 4st), and the Characteristics of the Four Phenotypes When Stockholm Patients Are Assigned to the Closest SENECA Phenotypes in Euclidean Space
**eTable 6.** Highlight Table of Pearson Correlation Coefficients Between Scaled Variable Values and Phenotype Assignment of the Oslo Cohort Using Phenotypes Derived From Each Site Where 26 Variables Were Available (Oslo, Oxford, Stockholm, SENECA)
**eTable 7.** Highlight Table of Pearson Correlation Coefficients Between Scaled Variable Values and Phenotype Assignment of the Oxford Cohort Using Phenotypes Derived From Sites Where 29 Variables Were Available (Oxford, Stockholm, SENECA)
**eTable 8.** Highlight Table of Pearson Correlation Coefficients Between Scaled Variable Values and Phenotype Assignment of the Stockholm Cohort Using Phenotypes Derived From Sites Where 29 Variables Were Available (Oxford, Stockholm, SENECA)
**eReference.**

This supplementary material has been provided by the authors to give readers additional information about their work.

© 2026 Yoon CH et al. *JAMA Network Open.*

**eFigure 1.** Flowchart of Patient Selection in the Oxford, Oslo and Stockholm Cohorts (Left: Oxford, Middle: Oslo, Right: Stockholm). Body fluid: blood, urine and/or cerebrospinal fluid. ED: Emergency Department, SOFA: Sequential Organ Failure Assessment.

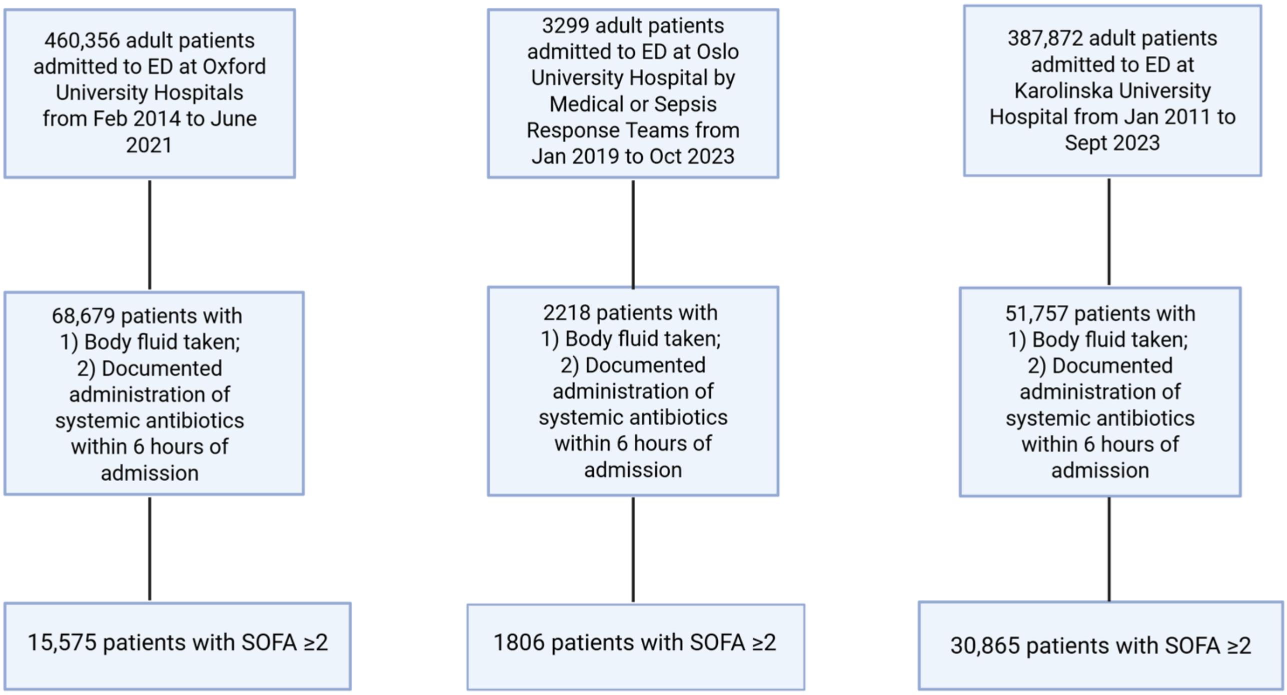


© 2026 Yoon CH et al. *JAMA Network Open.*

**eFigure 2.** Proportions of Missingness by Variable in the Oslo, Oxford and Stockholm Cohorts. SENECA derivation dataset missingness included in grey as a reference, numerical data extracted from Seymour et al [1]. Neutrophil granulocytes and urea were used instead of bands and blood urea nitrogen, respectively, in Oxford, Stockholm and Oslo. HR: Heart Rate, SBP: Systolic Blood Pressure, PaO2: partial pressure of oxygen, RR: Respiratory Rate, SaO2: oxygen saturation, GCS: Glasgow Coma Scale, INR: International Normalized Ratio, CRP: C-Reactive Protein, ESR: Erythrocyte Sedimentation Rate, ALAT: Alanine Transaminases, ASAT: Aspartate Transaminases, BUN: Blood Urea Nitrogen

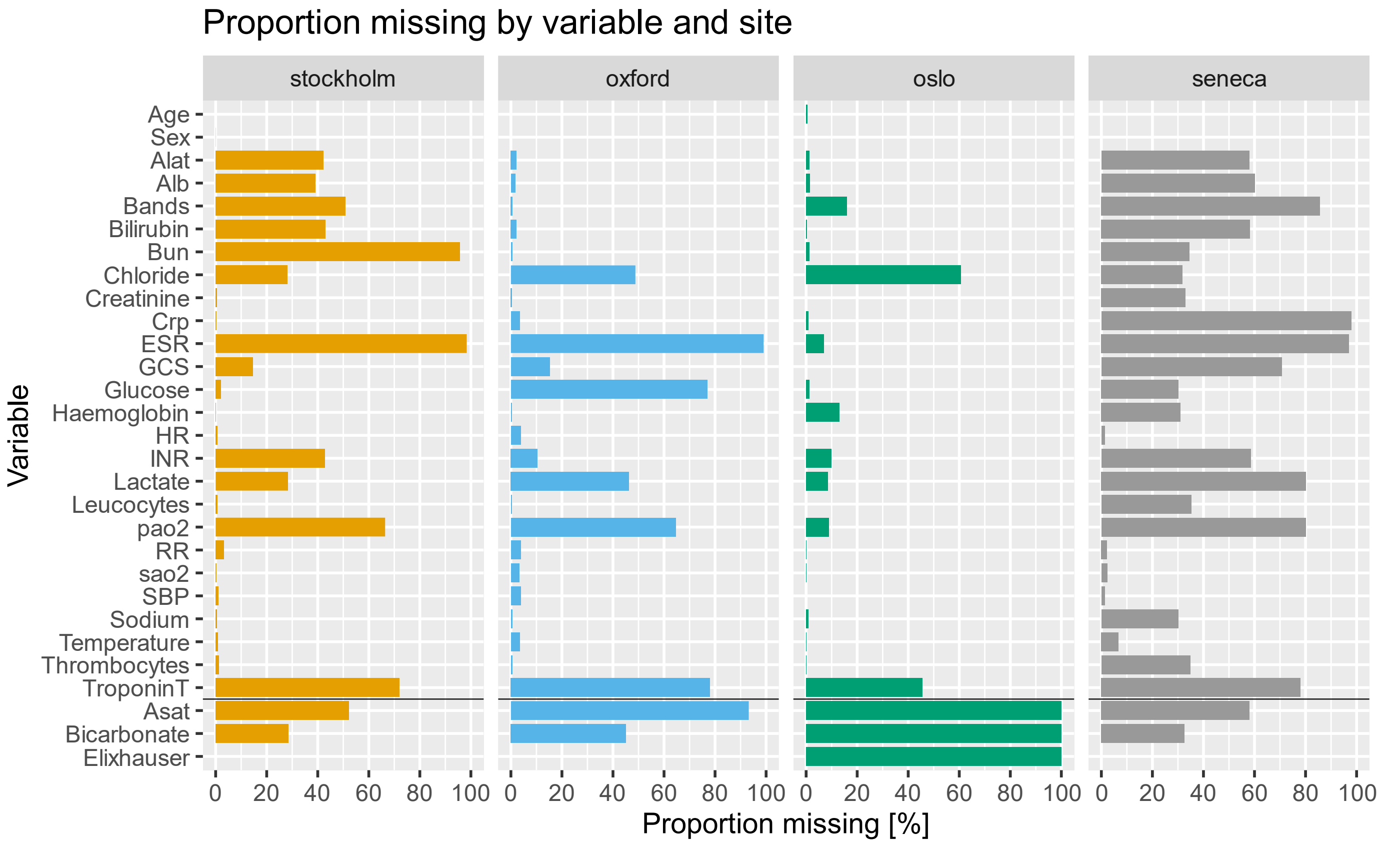


© 2026 Yoon CH et al. *JAMA Network Open.*

**eFigure 3.** Boxplots of Scaled Medians and IQR by Variable and Cohort. Neutrophil granulocytes and urea were used instead of bands and blood urea nitrogen, respectively, in Oxford, Stockholm and Oslo. HR: Heart Rate, SBP: Systolic Blood Pressure, PaO2: partial pressure of oxygen, RR: Respiratory Rate, SaO2: oxygen saturation, GCS: Glasgow Coma Scale, INR: International Normalized Ratio, CRP: C-Reactive Protein, ESR: Erythrocyte Sedimentation Rate, ALAT: Alanine Transaminases, ASAT: Aspartate Transaminases, BUN: Blood Urea Nitrogen

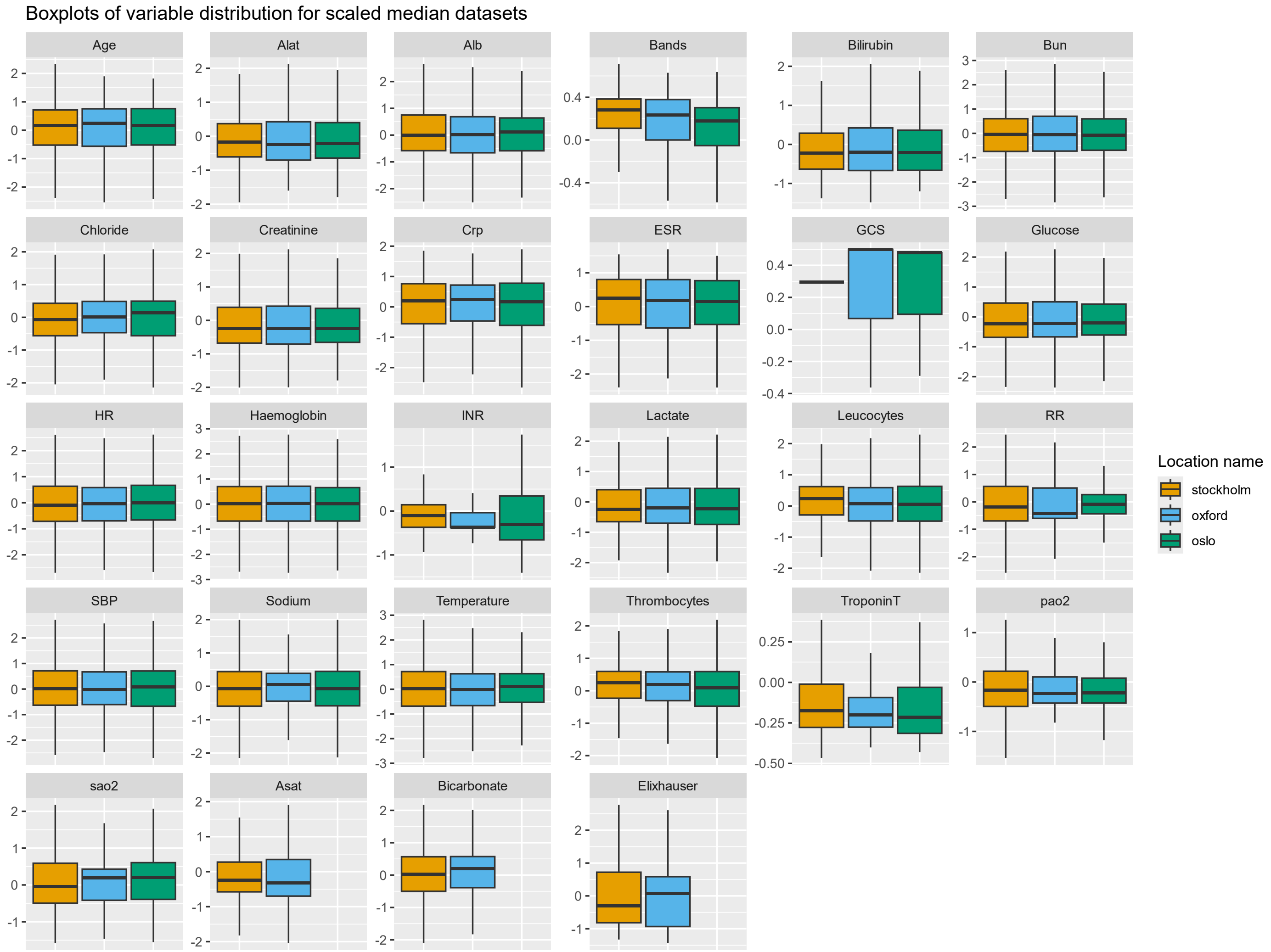


© 2026 Yoon CH et al. *JAMA Network Open.*

**eFigure 4.** Boxplots of Raw Medians and IQR by Variable and Cohort. Neutrophil granulocytes and urea were used instead of bands and blood urea nitrogen, respectively, in Oxford, Stockholm and Oslo. HR: Heart Rate, SBP: Systolic Blood Pressure, PaO2: partial pressure of oxygen, RR: Respiratory Rate, SaO2: oxygen saturation, GCS: Glasgow Coma Scale, INR: International Normalized Ratio, CRP: C-Reactive Protein, ESR: Erythrocyte Sedimentation Rate, ALAT: Alanine Transaminases, ASAT: Aspartate Transaminases, BUN: Blood Urea Nitrogen

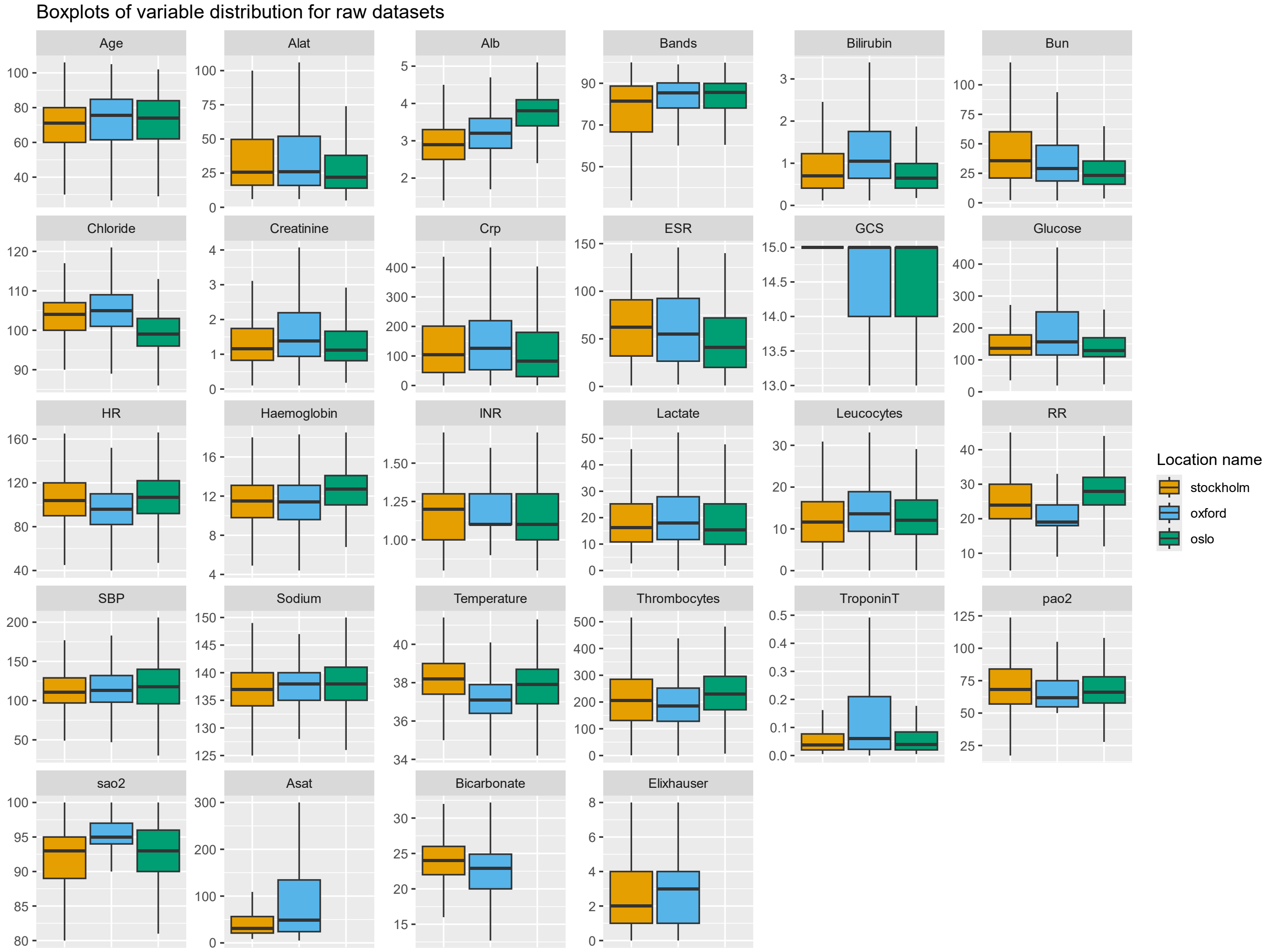


© 2026 Yoon CH et al. *JAMA Network Open.*

**eFigure 5.** Alluvial Plot Showing Differences in Patients From a Given Site Being Assigned to Phenotypes Derived From Individual Sites Incl. SENECA. In this example, patients are from the Oxford dataset with the common set of 26 variables. Left-most column is assigned with the centroid positions from the Oxford clustering results (i.e., calculated using the same dataset); meanwhile, 'oslo' indicates the assignment of Oxford patients to the centroids derived from the Oslo dataset based on the shortest Euclidean distance for each Oxford patient; similarly for 'stockholm' and 'seneca' with their respective site-derived centroids. Coloured according to Oxford phenotypes with phenotype ordering as close as possible to SENECA. Fleiss kappa for agreement is 0.49, and the percentage agreement between all is 43%. The Adjusted Rand Indices compared to Oxford are 0.32 Oslo, 0.54 Stockholm, and to 0.37 SENECA.

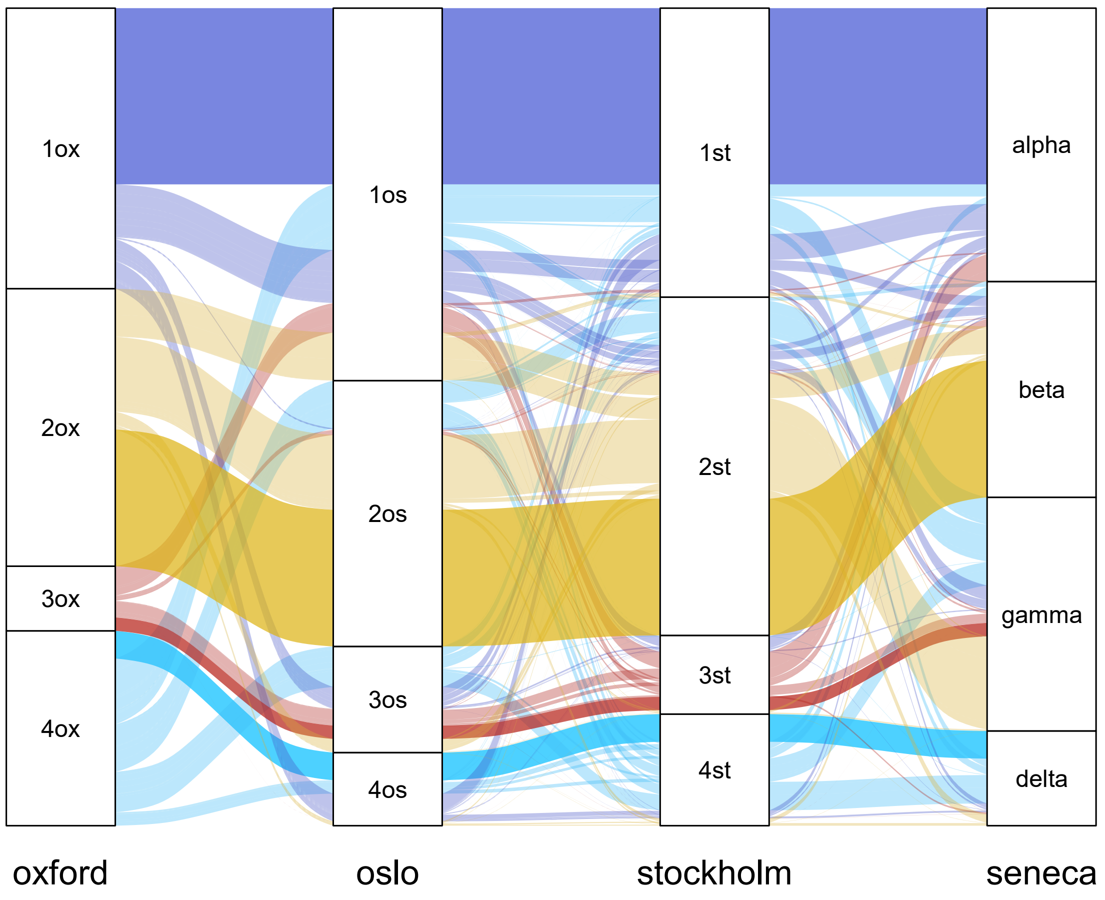


© 2026 Yoon CH et al. *JAMA Network Open.*

**eFigure 6.** Alluvial Plot Showing Differences in Patients From a Given Site Being Assigned to Phenotypes Derived From Individual Sites Incl. SENECA. In this example, patients are from the Oslo dataset with the common set of 26 variables. Left-most column is assigned with the centroid positions from the Oslo clustering result (i.e. calculated using the same dataset); meanwhile, 'oxford' indicates the assignment of Oslo patients to the centroids derived from the Oxford dataset based on the shortest Euclidean distance for each Oslo patient; similarly for 'stockholm' and 'seneca' with their respective site-derived centroids. Coloured according to Oslo phenotypes with phenotype ordering as close as possible to SENECA. Fleiss kappa for agreement is 0.50, and the percentage agreement between all is 45%. The Adjusted Rand Indices compared to Oxford are 0.33 Oslo, 0.37 Stockholm, and 0.27 SENECA.

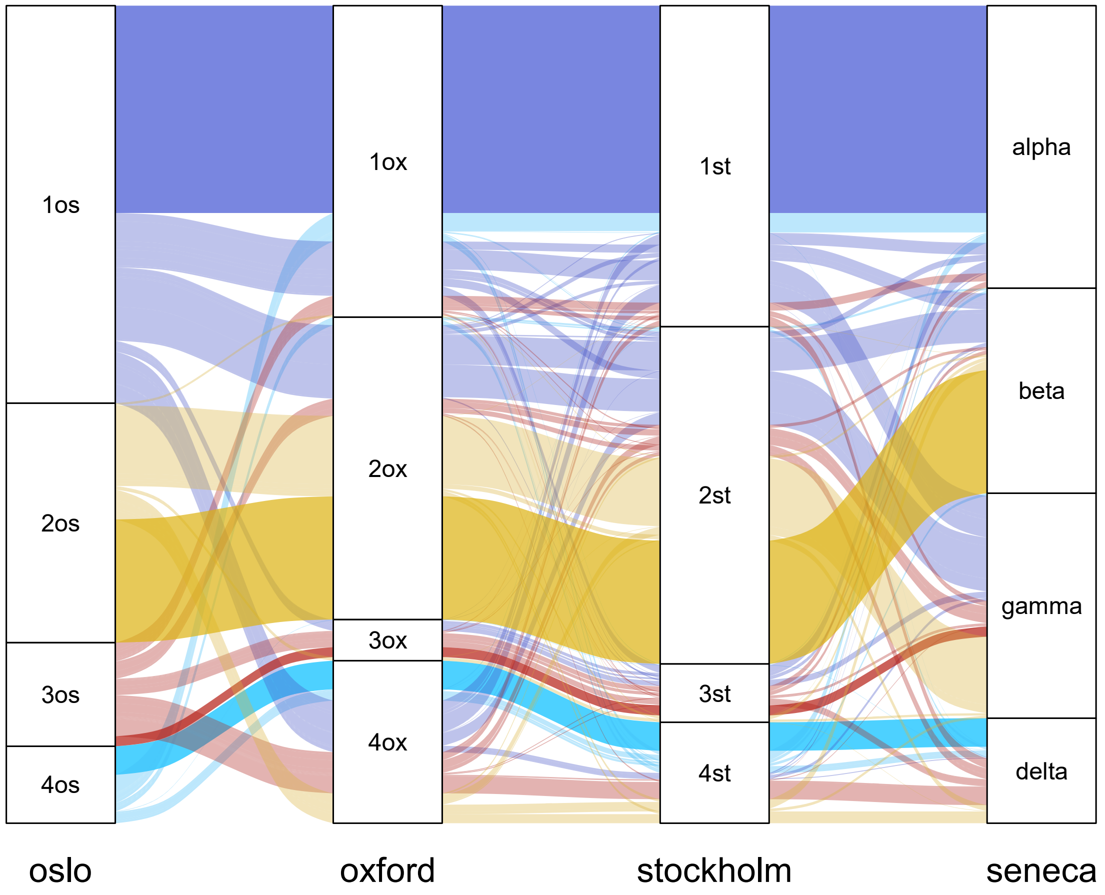


© 2026 Yoon CH et al. *JAMA Network Open.*

**eFigure 7.** Alluvial Plot Showing Differences in Patients From a Given Site Being Assigned to Phenotypes Derived From Individual Sites Incl. SENECA Using All 29 Variables. In this example, patients are from the Stockholm dataset. Left-most column is assigned with the centroid positions from the Stockholm clustering result (i.e. calculated using the same dataset), while the others are by assigning clusters using centroid information from the respective sites based on the shortest Euclidean distance for each Stockholm patient. Coloured according to Stockholm phenotypes with phenotype ordering as close as possible to SENECA. Fleiss kappa for agreement is 0.48, and the percentage agreement between all is 49%. The adjusted rand index is comparing Stockholm to Oxford 0.60, and to SENECA 0.25.

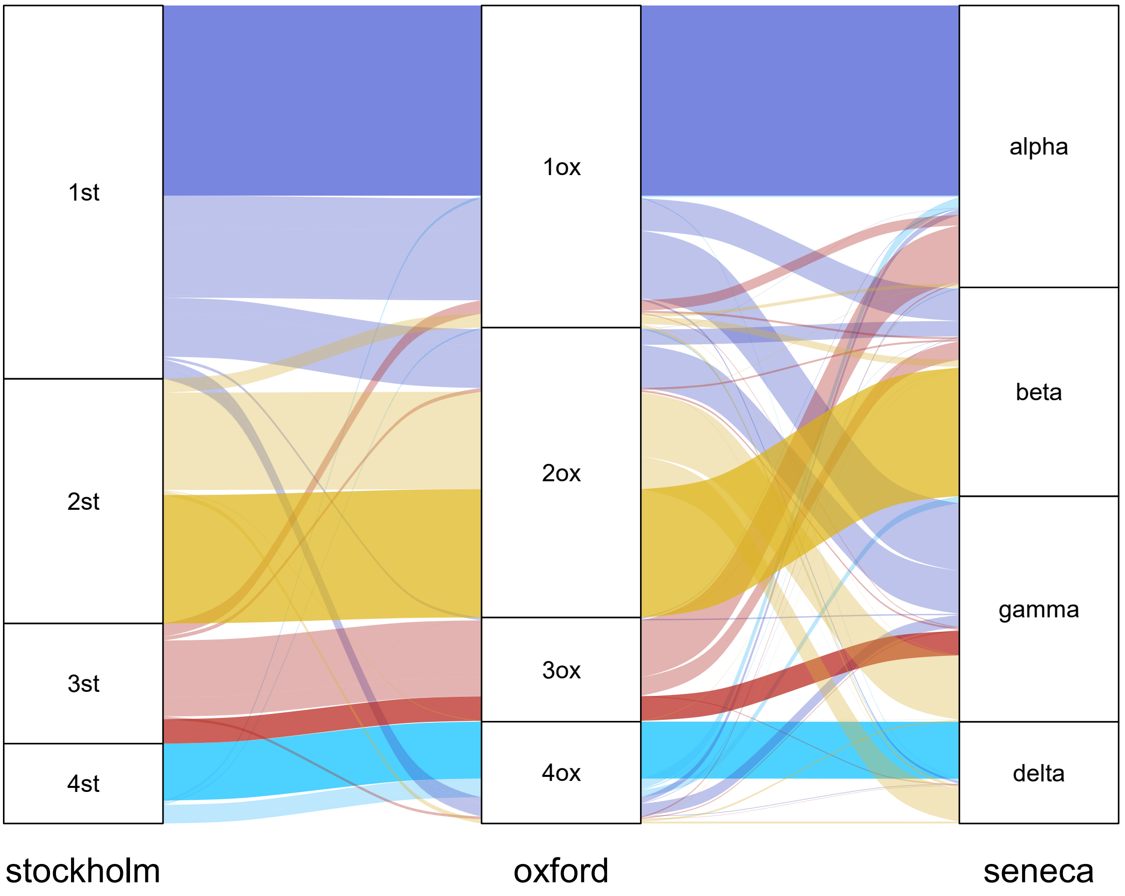


© 2026 Yoon CH et al. *JAMA Network Open.*

**eFigure 8.** Alluvial Plot Showing Differences in Patients From a Given Site Being Assigned to Phenotypes Derived From Individual Sites Incl. SENECA Using All 29 Variables. In this example, patients are from the Oxford dataset. Left-most column is assigned with the centroid positions from the Oxford clustering result (i.e. calculated using the same dataset), while the others are by assigning clusters using centroid information from the respective sites based on the shortest Euclidean distance for each Oxford patient. Coloured according to Oxford phenotypes with phenotype ordering as close as possible to SENECA. Fleiss kappa for agreement is 0.49, and the percentage agreement between all is 51%. The adjusted rand index comparing Oxford to Stockholm is 0.57, and to SENECA 0.26.

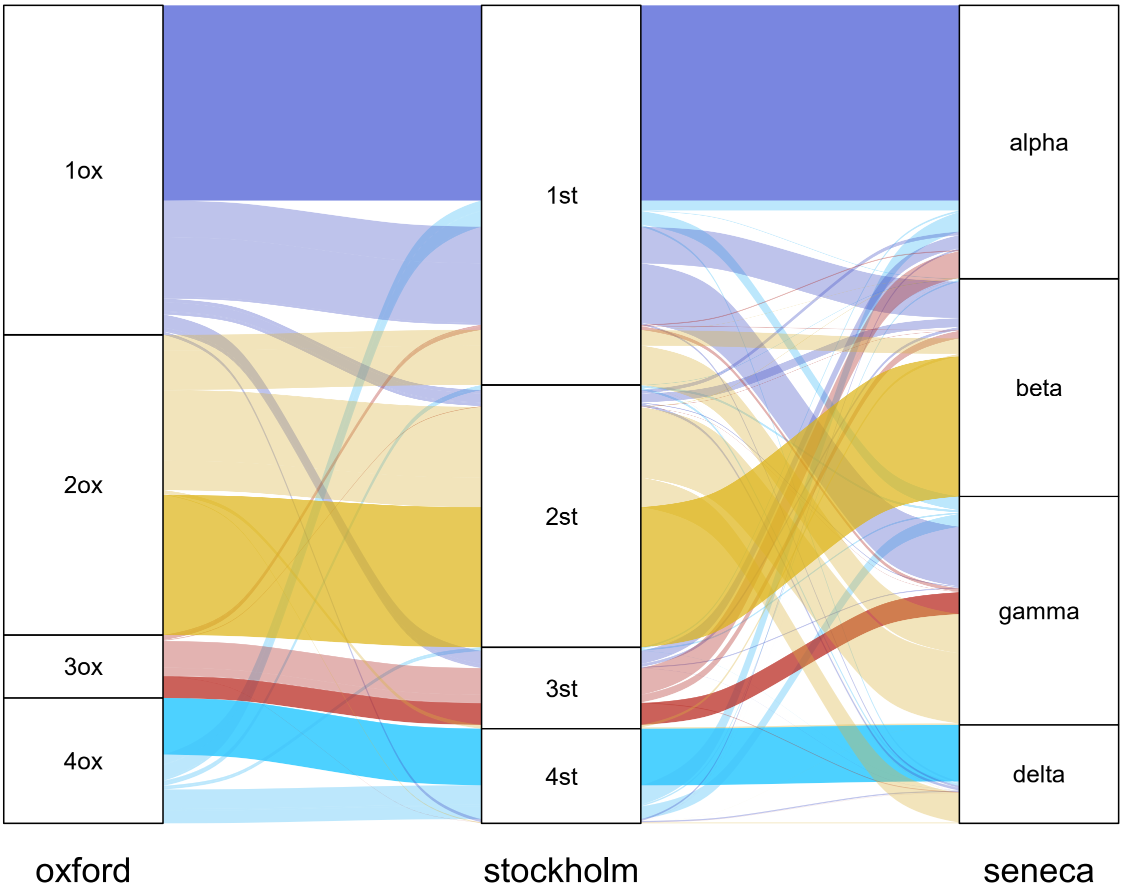


© 2026 Yoon CH et al. *JAMA Network Open.*

**eTable 1.** Variables and Their Units Included in the Analysis, as Originally Selected in the SENECA Data Analyzed by Seymour et al [1], and Using the Same Transformations. HR: Heart Rate, SBP: Systolic Blood Pressure, PaO2: partial pressure of oxygen, RR: Respiratory Rate, SpO2: oxygen saturation, GCS: Glasgow Coma Scale, INR: International Normalized Ratio, CRP: C-Reactive Protein, ESR: Erythrocyte Sedimentation Rate, ALAT: Alanine Transaminases, ASAT: Aspartate Transaminases, BUN: Blood Urea Nitrogen, IU: International Units.

| Variables | Units in SENECA data used by Seymour et al. | Transformation |
|---|---|---|
| Age | years | - |
| Sex | M=1, F=0 | - |
| HR | beats/min | - |
| SBP | mmHg | - |
| RR | breaths/min | - |
| PaO2 | mmHg | - |
| SpO2 | % [0-100] | Ln(101-sao2) |
| Temperature | °C | - |
| GCS | - | - |
| Elixhauser | - | - |
| Haemoglobin | g/dL | - |
| Leucocytes | $x10^9$/L | Ln |
| Bands | % [0-100] | Ln |
| Thrombocytes | $x10^9$/L | Ln |
| INR | - | Ln |
| CRP | mg/L | Ln |
| ESR | mm/h | Ln |
| ALAT | IU/L | Ln |
| ASAT | IU/L | Ln |
| Bilirubin | mg/dL | Ln |
| Albumin | g/dL | - |
| Creatinine | mg/dL | Ln |
| BUN | mg/dL | Ln |
| Chloride | mEq/L | - |
| Sodium | mEq/L | - |
| Bicarbonate | mEq/L | - |
| Lactate | mmol/L | Ln |
| Glucose | mg/dL | Ln |
| Troponin T | ng/mL | Ln |

© 2026 Yoon CH et al. *JAMA Network Open.*

**eTable 2.** Variables and Their Units in the SENECA, Stockholm, Oslo and Oxford Datasets. HR: Heart Rate, SBP: Systolic Blood Pressure, PaO2: partial pressure of oxygen, RR: Respiratory Rate, SpO2: oxygen saturation, GCS: Glasgow Coma Scale, INR: International Normalized Ratio, CRP: C-Reactive Protein, ESR: Erythrocyte Sedimentation Rate, ALAT: Alanine Transaminases, ASAT: Aspartate Transaminases, BUN: Blood Urea Nitrogen, IU: International Units. [a] Lactate was expressed as mmol/L for all calculations in accordance with the original methodology in Seymour et al. before being converted to mg/dL for wider clinical familiarity among readers [1].

| Variables | Units in SENECA data used by Seymour et al. | Variables in Stockholm, units | Variables in Oslo, units | Variables in Oxford, units |
|---|---|---|---|---|
| Age | years | years | Years | years |
| Sex | M=1, F=0 | M=1, F=0 | M=1, F=0 | M=1, F=0 |
| HR | beats/min | beats/min | beats/min | beats/min |
| SBP | mmHg | mmHg | mmHg | mmHg |
| RR | breaths/min | breaths/min | breaths/min | breaths/min |
| paO2 | mmHg | mmHg | mmHg | mmHg |
| SpO2 | % | % [0-100] | % [0-100] | % [0-100] |
| Temperature | °C | °C | °C | °C |
| GCS | - | - | - | - |
| Elixhauser | - | - | *Not Available* | - |
| Haemoglobin | g/dL | g/dL | g/dL | g/dL |
| Leucocytes | $x10^9$/L | $x10^9$/L | $x10^9$/L | $x10^9$/L |
| Bands (SENECA) / Neutrophils (other datasets) | % of leucocytes | % neutrophils/leucocytes | % neutrophils/leucocytes | % neutrophils/leucocytes |
| Thrombocytes | $x10^9$/L | $x10^9$/L | $x10^9$/L | $x10^9$/L |
| INR | - | - | - | - |
| CRP | mg/L | mg/L | mg/L | mg/L |
| ESR | mm/h | mm/h | mm/h | mm/h |
| ALAT | IU/L | IU/L | IU/L | IU/L |
| ASAT | IU/L | IU/L | *Not Available* | IU/L |
| Bilirubin | mg/dL | mg/dL | mg/dL | mg/dL |
| Albumin | g/dL | g/dL | g/dL | g/dL |

© 2026 Yoon CH et al. *JAMA Network Open.*

| | | | | |
|---|---|---|---|---|
| Creatinine | mg/dL | mg/dL | mg/dL | mg/dL |
| BUN (SENECA) / Urea (other datasets) | mg/dL | Urea mg/dL | Urea mg/dL | Urea mg/dL |
| Chloride | mEq/L (equivalent to mmol/L) | mmol/L | mmol/L | mmol/L |
| Sodium | mEq/L (equivalent to mmol/L) | mmol/L | mmol/L | mmol/L |
| Bicarbonate | mEq/L (equivalent to mmol/L) | mmol/L | *Not Available* | mmol/L |
| Lactate[a] | mmol/L | mmol/L | mmol/L | mmol/L |
| Glucose | mg/dL | mg/dL | mg/dL | mg/dL |
| Troponin T | ng/mL | ng/mL | ng/mL | ng/mL |

© 2026 Yoon CH et al. *JAMA Network Open.*

**eTable 3.** Characteristics of the Four Phenotypes Derived From Applying the Same Consensus Clustering Approach on the Oslo Cohort (1os, 2os, 3os, 4os), and the Characteristics of the Four Phenotypes When Oslo Patients Are Assigned to the Closest SENECA Phenotypes in Euclidean Space. Data source: Oslo, Variable set: 26v. Expressed as median [IQR], mean (SD), or no. (%). SBP: Systolic Blood Pressure, paO2: partial pressure of oxygen, SpO2: oxygen saturation, GCS: Glasgow Coma Scale, INR: International Normalized Ratio, CRP: C-Reactive Protein, ESR: Erythrocyte Sedimentation Rate, ALAT: Alanine Transaminases, ASAT: Aspartate Transaminases, BUN: Blood Urea Nitrogen, IU: International Units.

| | | **Phenotype** | | | | | | | |
|---|---|---|---|---|---|---|---|---|---|
| | | **oslo** | | | | **seneca** | | | |
| **Variable** | **Total** | **1os** | **2os** | **3os** | **4os** | **alpha** | **beta** | **gamma** | **delta** |
| No. of patients (%) | 1806 (100%) | 878 (49%) | 529 (29%) | 229 (13%) | 170 (9%) | 624 (35%) | 453 (25%) | 497 (28%) | 232 (13%) |
| Age, years | 71 (17) | 69 (18) | 77 (12) | 66 (17) | 68 (18) | 66 (19) | 78 (12) | 70 (16) | 72 (15) |
| Sex, male no. (%) | 1068 (59%) | 478 (54%) | 320 (60%) | 164 (72%) | 106 (62%) | 351 (56%) | 283 (62%) | 273 (55%) | 161 (69%) |
| ALAT, IU/L | 22 [15 - 38] | 20 [14 - 30] | 18 [13 - 30] | 49 [28 - 125] | 34 [22 - 57] | 22 [16 - 34] | 17 [12 - 27] | 21 [14 - 35] | 53 [29 - 143] |
| Albumin, g/dL | 3.7 (0.57) | 4.0 (0.43) | 3.4 (0.45) | 3.3 (0.57) | 3.9 (0.63) | 4.2 (0.41) | 3.6 (0.47) | 3.4 (0.48) | 3.4 (0.55) |
| Bands, % | 86 [79 - 89] | 85 [79 - 89] | 87 [81 - 90] | 87 [79 - 92] | 83 [76 - 88] | 84 [77 - 89] | 85 [79 - 89] | 87 [82 - 90] | 86 [81 - 91] |
| Bilirubin, mg/dL | 0.64 [0.41 - 0.99] | 0.64 [0.41 - 0.94] | 0.53 [0.35 - 0.76] | 1.5 [0.88 - 2.7] | 0.53 [0.35 - 0.94] | 0.64 [0.41 - 0.99] | 0.53 [0.35 - 0.76] | 0.64 [0.47 - 0.94] | 0.99 [0.58 - 1.9] |
| BUN, mg/dL | 23 [16 - 35] | 17 [13 - 23] | 37 [27 - 55] | 26 [18 - 39] | 27 [17 - 39] | 16 [12 - 22] | 36 [27 - 55] | 21 [15 - 30] | 37 [26 - 54] |
| Chloride, mmol/L | 99 (5.7) | 99 (4.5) | 100 (6.1) | 97 (5.8) | 101 (8.5) | 100 (4.7) | 100 (5.6) | 97 (5.0) | 101 (8.0) |
| Creatinine, mg/dL | 1.1 [0.81 - 1.7] | 0.89 [0.71 - 1.2] | 1.7 [1.2 - 2.6] | 1.2 [0.96 - 1.8] | 1.2 [0.90 - 1.9] | 0.88 [0.69 - 1.1] | 1.7 [1.3 - 2.7] | 1.0 [0.77 - 1.4] | 1.7 [1.2 - 2.3] |
| CRP, mg/L | 82 [30 - 180] | 58 [22 - 133] | 139 [69 - 239] | 124 [56 - 235] | 27 [5.9 - 79] | 30 [12 - 81] | 91 [38 - 175] | 178 [100 - 280] | 81 [36 - 186] |
| ESR, mm/h | 40 [21 - 71] | 34 [18 - 57] | 68 [43 - 99] | 38 [16 - 65] | 16 [7.3 - 33] | 21 [11 - 35] | 59 [38 - 89] | 65 [44 - 95] | 26 [11 - 53] |
| GCS | 14 (2.6) | 14 (1.3) | 14 (1.5) | 14 (2.1) | 8.5 (4.5) | 14 (2.7) | 14 (1.5) | 14 (1.5) | 11 (4.2) |
| Glucose, mg/dL | 130 [110 - 169] | 128 [110 - 159] | 126 [106 - 160] | 132 [108 - 173] | 195 [146 - 268] | 126 [110 - 162] | 128 [108 - 166] | 133 [110 - 168] | 147 [114 - 200] |
| Hemoglobin, g/dL | 13 (2.2) | 13 (1.7) | 11 (1.7) | 13 (2.4) | 14 (2.3) | 14 (1.6) | 11 (1.8) | 12 (1.9) | 13 (2.7) |
| Heart rate, 1/min | 107 (23) | 109 (20) | 99 (21) | 116 (24) | 111 (27) | 107 (20) | 94 (20) | 116 (20) | 113 (26) |
| INR | 1.1 [1.0 - 1.3] | 1.1 [1.0 - 1.2] | 1.1 [1.0 - 1.2] | 1.3 [1.2 - 1.5] | 1.1 [1.0 - 1.2] | 1.1 [1.0 - 1.2] | 1.1 [1.0 - 1.2] | 1.2 [1.1 - 1.3] | 1.2 [1.1 - 1.4] |
| Lactate, mg/dL | 15 [10 - 24] | 13 [9.0 - 18] | 14 [9.9 - 21] | 29 [20 - 44] | 34 [19 - 56] | 13 [9.0 - 21] | 13 [9.0 - 18] | 16 [12 - 24] | 36 [23 - 60] |
| Leucocytes, x10^3/µL | 12 [8.7 - 17] | 12 [8.9 - 16] | 13 [9.3 - 18] | 8.8 [3.7 - 14] | 15 [11 - 20] | 11 [8.3 - 15] | 12 [8.5 - 16] | 14 [9.3 - 19] | 14 [8.6 - 20] |
| paO2, mmHg | 74 (38) | 66 (22) | 72 (24) | 75 (29) | 121 (87) | 72 (34) | 72 (22) | 66 (22) | 100 (74) |
| Respiratory rate, 1/min | 29 (11) | 29 (9.8) | 27 (7.0) | 31 (9.1) | 31 (25) | 28 (7.3) | 26 (6.4) | 32 (18) | 30 (8.9) |
| SpO2, % | 93 [90 - 96] | 93 [90 - 95] | 94 [90 - 97] | 94 [90 - 97] | 94 [89 - 99] | 93 [90 - 96] | 94 [91 - 97] | 93 [89 - 95] | 94 [88 - 97] |
| SBP, mmHg | 118 [96 - 140] | 130 [110 - 150] | 104 [89 - 125] | 99 [86 - 120] | 108 [85 - 134] | 130 [109 - 151] | 118 [98 - 139] | 111 [93 - 132] | 96 [80 - 115] |

© 2026 Yoon CH et al. *JAMA Network Open.*

| Variable | Total | Phenotype | | | | | | | |
|---|---|---|---|---|---|---|---|---|---|
| | | oslo | | | | seneca | | | |
| | | 1os | 2os | 3os | 4os | alpha | beta | gamma | delta |
| Sodium, mmol/L | 138 (5.8) | 138 (4.5) | 139 (6.5) | 136 (5.4) | 141 (8.5) | 139 (4.6) | 139 (5.9) | 136 (5.1) | 141 (8.2) |
| Temperature, °C | 37.7 (1.5) | 38.2 (1.1) | 37.4 (1.4) | 37.8 (1.5) | 36.2 (2.5) | 38.0 (1.4) | 37.5 (1.6) | 38.1 (1.2) | 36.8 (2.0) |
| Thrombocytes, x10^9/L | 229 [171 - 296] | 226 [178 - 284] | 259 [189 - 331] | 147 [95 - 207] | 258 [211 - 324] | 219 [169 - 268] | 238 [180 - 304] | 245 [183 - 327] | 204 [139 - 288] |
| Troponin, ng/mL | 0.037 [0.022 - 0.065] | 0.027 [0.018 - 0.040] | 0.056 [0.036 - 0.094] | 0.045 [0.024 - 0.069] | 0.051 [0.028 - 0.099] | 0.026 [0.015 - 0.037] | 0.050 [0.033 - 0.084] | 0.037 [0.024 - 0.061] | 0.070 [0.045 - 0.18] |
| 28-Day mortality no. (%) | 337 (19%) | 77 (9%) | 133 (25%) | 68 (30%) | 59 (35%) | 60 (10%) | 86 (19%) | 89 (18%) | 102 (44%) |
| 365-Day mortality no. (%) | 681 (38%) | 225 (26%) | 270 (51%) | 106 (46%) | 80 (47%) | 134 (21%) | 211 (47%) | 198 (40%) | 138 (59%) |

© 2026 Yoon CH et al. *JAMA Network Open.*

**eTable 4.** Characteristics of the Four Phenotypes Derived From Applying the Same Consensus Clustering Approach on the Oxford Cohort (1ox, 2ox, 3ox, 4ox), and the Characteristics of the Four Phenotypes When Oxford Patients Are Assigned to the Closest SENECA Phenotypes in Euclidean Space. Data source: Oxford, Variable set: 29v. Expressed as median [IQR], mean (SD), or no. (%). SBP: Systolic Blood Pressure, paO2: partial pressure of oxygen, SpO2: oxygen saturation, GCS: Glasgow Coma Scale, INR: International Normalized Ratio, CRP: C-Reactive Protein, ESR: Erythrocyte Sedimentation Rate, ALAT: Alanine Transaminases, ASAT: Aspartate Transaminases, BUN: Blood Urea Nitrogen, IU: International Units.

| | | **Phenotype** | | | | | | | |
|---|---|---|---|---|---|---|---|---|---|
| | | **oxford** | | | | **seneca** | | | |
| **Variable** | **Total** | **1ox** | **2ox** | **3ox** | **4ox** | **alpha** | **beta** | **gamma** | **delta** |
| No. of patients (%) | 15575 (100%) | 6273 (40%) | 5715 (37%) | 1198 (8%) | 2389 (15%) | 5206 (33%) | 4145 (27%) | 4348 (28%) | 1876 (12%) |
| Age, years | 71 (18) | 71 (19) | 76 (14) | 61 (18) | 68 (18) | 66 (20) | 78 (14) | 72 (16) | 72 (17) |
| Sex, male no. (%) | 9067 (58%) | 3665 (58%) | 3425 (60%) | 637 (53%) | 1340 (56%) | 3067 (59%) | 2483 (60%) | 2377 (55%) | 1140 (61%) |
| ALAT, IU/L | 26 [16 - 51] | 22 [15 - 34] | 22 [15 - 37] | 31 [19 - 54] | 157 [82 - 309] | 26 [17 - 49] | 19 [13 - 30] | 26 [17 - 47] | 99 [42 - 279] |
| Albumin, g/dL | 3.2 (0.59) | 3.4 (0.49) | 3 (0.57) | 3.2 (0.58) | 3.1 (0.64) | 3.5 (0.46) | 3.1 (0.54) | 2.9 (0.58) | 3.1 (0.62) |
| Bands, % | 85 [78 - 90] | 85 [78 - 89] | 87 [82 - 91] | 59 [23 - 77] | 87 [81 - 91] | 83 [74 - 89] | 84 [77 - 89] | 87 [82 - 91] | 88 [83 - 92] |
| Bilirubin, mg/dL | 1.1 [0.64 - 1.8] | 1.1 [0.64 - 1.6] | 0.76 [0.53 - 1.2] | 1.1 [0.64 - 1.6] | 2.9 [1.6 - 5.1] | 1.2 [0.70 - 2] | 0.70 [0.47 - 1.1] | 1.1 [0.70 - 1.7] | 1.8 [0.88 - 3.8] |
| BUN, mg/dL | 29 [18 - 49] | 21 [15 - 29] | 52 [37 - 74] | 20 [14 - 29] | 25 [17 - 39] | 18 [13 - 24] | 52 [37 - 75] | 29 [21 - 42] | 40 [25 - 64] |
| Chloride, mmol/L | 105 (6.3) | 104 (5.7) | 106 (7.2) | 103 (4.3) | 104 (6.1) | 104 (5.2) | 105 (6.4) | 103 (5.7) | 109 (8.7) |
| Creatinine, mg/dL | 1.4 [0.94 - 2.2] | 1.1 [0.81 - 1.4] | 2.3 [1.6 - 3.7] | 0.98 [0.76 - 1.3] | 1.2 [0.86 - 1.8] | 0.96 [0.76 - 1.3] | 2.4 [1.7 - 4.1] | 1.3 [0.94 - 1.9] | 1.7 [1.2 - 2.8] |
| CRP, mg/L | 124 [53 - 217] | 78 [24 - 160] | 177 [102 - 266] | 123 [62 - 213] | 125 [61 - 210] | 59 [19 - 124] | 119 [58 - 201] | 211 [142 - 295] | 137 [68 - 220] |
| ESR, mm/h | 41 [20 - 72] | 23 [11 - 43] | 70 [43 - 88] | 57 [35 - 85] | 35 [16 - 61] | 19 [11 - 35] | 57 [36 - 85] | 68 [43 - 86] | 30 [14 - 55] |
| GCS | 14 (2.3) | 13 (2.7) | 14 (2.1) | 15 (0.92) | 14 (2.1) | 14 (2.6) | 14 (1.5) | 14 (1.9) | 13 (3.4) |
| Glucose, mg/dL | 142 [122 - 184] | 133 [119 - 159] | 166 [133 - 225] | 124 [112 - 146] | 142 [121 - 180] | 128 [115 - 151] | 153 [126 - 202] | 150 [124 - 193] | 159 [128 - 226] |
| Hemoglobin, g/dL | 11 (2.5) | 13 (2) | 10 (2.1) | 8.4 (2.1) | 12 (2.4) | 13 (2.3) | 10 (2.1) | 10 (2.3) | 11 (2.6) |
| Heart rate, 1/min | 97 (21) | 93 (21) | 97 (21) | 101 (19) | 103 (22) | 94 (19) | 86 (17) | 106 (20) | 107 (24) |
| INR | 1.1 [1.1 - 1.3] | 1.1 [1.0 - 1.2] | 1.2 [1.1 - 1.3] | 1.1 [1.1 - 1.2] | 1.2 [1.1 - 1.6] | 1.1 [1.0 - 1.2] | 1.1 [1.1 - 1.3] | 1.2 [1.1 - 1.3] | 1.3 [1.1 - 1.8] |
| Lactate, mg/dL | 17 [14 - 24] | 16 [13 - 21] | 18 [14 - 25] | 14 [12 - 17] | 24 [17 - 37] | 16 [13 - 21] | 15 [12 - 19] | 18 [14 - 25] | 35 [24 - 54] |
| Leucocytes, x10^3/µL | 14 [9.4 - 19] | 13 [9.2 - 17] | 15 [11 - 21] | 5.0 [1.9 - 11] | 15 [11 - 21] | 12 [8.0 - 16] | 13 [9.0 - 17] | 16 [11 - 22] | 17 [12 - 23] |
| paO2, mmHg | 66 (19) | 66 (18) | 67 (22) | 65 (15) | 66 (17) | 66 (17) | 67 (20) | 64 (13) | 71 (31) |
| Respiratory rate, 1/min | 21 (5.4) | 21 (5.1) | 22 (5.8) | 20 (3.9) | 22 (5.8) | 20 (4.3) | 20 (3.6) | 23 (6) | 24 (7.1) |
| SpO2, % | 95 [94 - 97] | 95 [94 - 97] | 95 [93 - 97] | 96 [94 - 97] | 95 [94 - 97] | 96 [94 - 98] | 96 [94 - 97] | 94 [92 - 96] | 95 [93 - 97] |
| SBP, mmHg | 113 [99 - 132] | 121 [105 - 140] | 108 [94 - 126] | 108 [97 - 120] | 110 [97 - 125] | 122 [108 - 140] | 119 [104 - 138] | 102 [91 - 116] | 104 [90 - 120] |
| Sodium, mmol/L | 138 (6.0) | 138 (5.3) | 138 (7) | 136 (4.1) | 136 (5.6) | 138 (5.0) | 138 (6.1) | 137 (5.4) | 140 (8.5) |

© 2026 Yoon CH et al. *JAMA Network Open.*

| Variable | Total | Phenotype | | | | | | | |
|---|---|---|---|---|---|---|---|---|---|
| | | oxford | | | | seneca | | | |
| | | 1ox | 2ox | 3ox | 4ox | alpha | beta | gamma | delta |
| Temperature, °C | 37.1 (1.1) | 37.1 (1.1) | 37.0 (1.1) | 37.7 (1.0) | 37.1 (1.1) | 37.2 (1.0) | 36.7 (1.0) | 37.5 (1.0) | 36.9 (1.2) |
| Thrombocytes, x10^9/L | 186 [128 - 252] | 192 [141 - 251] | 207 [151 - 275] | 40 [13 - 74] | 175 [117 - 239] | 177 [122 - 238] | 205 [149 - 271] | 184 [120 - 257] | 175 [116 - 239] |
| Troponin, ng/mL | 0.059 [0.039 - 0.090] | 0.050 [0.032 - 0.080] | 0.064 [0.040 - 0.11] | 0.041 [0.029 - 0.060] | 0.060 [0.040 - 0.11] | 0.050 [0.030 - 0.080] | 0.060 [0.040 - 0.098] | 0.052 [0.037 - 0.085] | 0.090 [0.050 - 0.29] |
| ASAT, IU/L | 27 [19 - 50] | 24 [19 - 33] | 24 [18 - 34] | 27 [19 - 44] | 160 [87 - 300] | 27 [20 - 48] | 21 [18 - 30] | 27 [19 - 43] | 124 [51 - 291] |
| Bicarbonate, mmol/L | 22 (3.7) | 24 (2.7) | 21 (4.1) | 24 (2.4) | 22 (4) | 24 (2.3) | 22 (3.9) | 23 (3.2) | 19 (4.8) |
| Elixhauser | 2.8 (2) | 2.2 (1.7) | 3.7 (2.1) | 2.2 (1.7) | 2.8 (1.9) | 2.0 (1.6) | 3.8 (2.1) | 2.8 (1.8) | 3.1 (1.9) |
| 28-Day mortality no. (%) | 1875 (12%) | 590 (9%) | 832 (15%) | 107 (9%) | 346 (14%) | 372 (7%) | 599 (14%) | 518 (12%) | 386 (21%) |
| 365-Day mortality no. (%) | 4377 (28%) | 1263 (20%) | 1944 (34%) | 429 (36%) | 741 (31%) | 884 (17%) | 1421 (34%) | 1380 (32%) | 692 (37%) |

© 2026 Yoon CH et al. *JAMA Network Open.*

**eTable 5.** Characteristics of the Four Phenotypes Derived From Applying the Same Consensus Clustering Approach on the Stockholm Cohort (1st, 2st, 3st, 4st), and the Characteristics of the Four Phenotypes When Stockholm Patients Are Assigned to the Closest SENECA Phenotypes in Euclidean Space. Data source: Stockholm, Variable set: 29v. Expressed as median [IQR], mean (SD), or no. (%). SBP: Systolic Blood Pressure, paO2: partial pressure of oxygen, SpO2: oxygen saturation, GCS: Glasgow Coma Scale, INR: International Normalized Ratio, CRP: C-Reactive Protein, ESR: Erythrocyte Sedimentation Rate, ALAT: Alanine Transaminases, ASAT: Aspartate Transaminases, BUN: Blood Urea Nitrogen, IU: International Units.

| | | **Phenotype** | | | | | | | |
|---|---|---|---|---|---|---|---|---|---|
| | | **stockholm** | | | | **seneca** | | | |
| **Variable** | **Total** | **1st** | **2st** | **3st** | **4st** | **alpha** | **beta** | **gamma** | **delta** |
| No. of patients (%) | 30865 (100%) | 14083 (46%) | 9231 (30%) | 4537 (15%) | 3014 (10%) | 10637 (34%) | 7878 (26%) | 8512 (28%) | 3838 (12%) |
| Age, years | 68 (16) | 67 (16) | 76 (12) | 58 (16) | 68 (16) | 62 (18) | 75 (13) | 69 (14) | 73 (15) |
| Sex, male no. (%) | 18184 (59%) | 7999 (57%) | 5776 (63%) | 2551 (56%) | 1858 (62%) | 6427 (60%) | 4922 (62%) | 4438 (52%) | 2397 (62%) |
| ALAT, IU/L | 25 [18 - 38] | 23 [18 - 33] | 21 [16 - 28] | 30 [20 - 46] | 107 [59 - 224] | 28 [20 - 40] | 20 [16 - 27] | 23 [17 - 32] | 60 [31 - 146] |
| Albumin, g/dL | 2.9 (0.53) | 3.1 (0.47) | 2.6 (0.49) | 2.9 (0.48) | 2.8 (0.60) | 3.3 (0.38) | 2.8 (0.42) | 2.6 (0.45) | 2.7 (0.57) |
| Bands, % | 82 [76 - 86] | 83 [78 - 86] | 83 [79 - 87] | 50 [25 - 81] | 84 [80 - 87] | 81 [72 - 85] | 82 [76 - 86] | 83 [78 - 87] | 84 [80 - 87] |
| Bilirubin, mg/dL | 0.70 [0.47 - 1.1] | 0.70 [0.53 - 0.94] | 0.58 [0.41 - 0.82] | 0.70 [0.53 - 0.99] | 2.2 [1.2 - 4.1] | 0.76 [0.53 - 1.1] | 0.58 [0.41 - 0.82] | 0.64 [0.47 - 0.94] | 1.2 [0.64 - 2.6] |
| BUN, mg/dL | 25 [17 - 36] | 20 [15 - 26] | 42 [33 - 55] | 17 [14 - 23] | 27 [20 - 37] | 17 [13 - 21] | 38 [29 - 52] | 24 [18 - 30] | 34 [25 - 49] |
| Chloride, mmol/L | 103 (6.1) | 102 (4.9) | 106 (7.6) | 102 (4.0) | 103 (6.0) | 103 (4.6) | 104 (6.1) | 102 (5.6) | 107 (8.6) |
| Creatinine, mg/dL | 1.2 [0.83 - 1.7] | 0.98 [0.76 - 1.3] | 2.0 [1.4 - 3.1] | 0.85 [0.69 - 1.1] | 1.2 [0.87 - 1.8] | 0.89 [0.71 - 1.1] | 1.9 [1.3 - 3] | 1.0 [0.77 - 1.4] | 1.6 [1.1 - 2.5] |
| CRP, mg/L | 104 [44 - 201] | 96 [36 - 190] | 145 [73 - 244] | 73 [36 - 142] | 90 [33 - 180] | 48 [18 - 104] | 118 [63 - 203] | 187 [111 - 282] | 101 [40 - 200] |
| ESR, mm/h | 55 [33 - 83] | 49 [28 - 78] | 70 [43 - 88] | 61 [39 - 84] | 36 [19 - 61] | 35 [22 - 52] | 72 [48 - 88] | 83 [62 - 95] | 34 [20 - 56] |
| GCS | 15 (1.7) | 15 (1.4) | 14 (2.1) | 15 (0.56) | 14 (2.1) | 15 (1.4) | 15 (1.0) | 15 (1.1) | 13 (3.2) |
| Glucose, mg/dL | 137 [115 - 178] | 137 [117 - 169] | 153 [123 - 211] | 123 [108 - 144] | 142 [117 - 191] | 130 [112 - 157] | 141 [117 - 186] | 142 [119 - 184] | 159 [124 - 220] |
| Hemoglobin, g/dL | 11 (2.3) | 13 (1.9) | 11 (2.0) | 9.1 (1.8) | 12 (2.4) | 12 (2.4) | 11 (2) | 11 (2.1) | 12 (2.5) |
| Heart rate, 1/min | 106 (22) | 107 (21) | 104 (24) | 104 (20) | 108 (25) | 104 (19) | 93 (18) | 117 (21) | 113 (26) |
| INR | 1.1 [1.1 - 1.3] | 1.1 [1.1 - 1.2] | 1.2 [1.1 - 1.3] | 1.1 [1.0 - 1.2] | 1.3 [1.2 - 1.7] | 1.1 [1.0 - 1.2] | 1.1 [1.1 - 1.2] | 1.2 [1.1 - 1.3] | 1.3 [1.1 - 1.6] |
| Lactate, mg/dL | 14 [11 - 23] | 14 [9.9 - 18] | 17 [12 - 28] | 12 [9.9 - 15] | 27 [16 - 47] | 13 [9.9 - 17] | 13 [9.9 - 17] | 16 [12 - 23] | 34 [22 - 56] |
| Leucocytes, x10^3/µL | 12 [7.0 - 16] | 12 [8.9 - 16] | 14 [9.6 - 19] | 0.70 [0.20 - 2.2] | 12 [8.3 - 18] | 9.8 [4.1 - 14] | 12 [8.2 - 16] | 13 [7.7 - 18] | 14 [9.1 - 19] |
| paO2, mmHg | 77 (27) | 69 (18) | 82 (33) | 85 (22) | 85 (40) | 74 (21) | 80 (25) | 71 (20) | 90 (48) |
| Respiratory rate, 1/min | 26 (7.9) | 26 (7.8) | 26 (8.3) | 21 (5.3) | 26 (8.3) | 24 (7.1) | 23 (6.0) | 29 (8.2) | 29 (9.0) |
| SpO2, % | 93 [89 - 95] | 91 [88 - 94] | 93 [88 - 95] | 96 [94 - 98] | 94 [90 - 96] | 93 [90 - 95] | 94 [91 - 96] | 91 [87 - 94] | 92 [87 - 95] |
| SBP, mmHg | 111 [97 - 129] | 116 [100 - 131] | 105 [90 - 123] | 113 [100 - 126] | 108 [92 - 125] | 119 [104 - 132] | 118 [101 - 134] | 104 [90 - 120] | 100 [85 - 118] |
| Sodium, mmol/L | 137 (5.8) | 137 (4.6) | 139 (7.6) | 136 (3.9) | 136 (5.9) | 138 (4.3) | 138 (5.9) | 136 (5.2) | 140 (8.8) |

© 2026 Yoon CH et al. *JAMA Network Open.*

| | | Phenotype | | | | | | | |
|---|---|---|---|---|---|---|---|---|---|
| | | stockholm | | | | seneca | | | |
| **Variable** | **Total** | **1st** | **2st** | **3st** | **4st** | **alpha** | **beta** | **gamma** | **delta** |
| Temperature, °C | 38.2 (1.1) | 38.5 (1.0) | 37.7 (1.2) | 38.5 (0.9) | 37.9 (1.3) | 38.5 (1.0) | 37.8 (1.1) | 38.4 (1.0) | 37.6 (1.4) |
| Thrombocytes, x10^9/L | 205 [131 - 284] | 224 [165 - 296] | 236 [171 - 318] | 32 [12 - 78] | 194 [129 - 269] | 175 [97 - 247] | 218 [151 - 295] | 232 [148 - 327] | 204 [136 - 281] |
| Troponin, ng/mL | 0.032 [0.022 - 0.049] | 0.027 [0.020 - 0.038] | 0.047 [0.033 - 0.070] | 0.024 [0.017 - 0.032] | 0.042 [0.026 - 0.075] | 0.024 [0.017 - 0.033] | 0.039 [0.028 - 0.058] | 0.033 [0.023 - 0.046] | 0.057 [0.035 - 0.10] |
| ASAT, IU/L | 30 [23 - 44] | 29 [23 - 38] | 29 [23 - 40] | 25 [19 - 33] | 148 [87 - 281] | 29 [23 - 40] | 26 [21 - 34] | 30 [23 - 41] | 93 [48 - 216] |
| Bicarbonate, mmol/L | 24 (3.7) | 25 (2.8) | 22 (4.1) | 25 (2.2) | 22 (4.6) | 25 (2.4) | 23 (3.6) | 25 (3.4) | 20 (4.8) |
| Elixhauser | 2.6 (2) | 2 (1.6) | 3.7 (2.2) | 2.1 (1.4) | 2.7 (1.9) | 1.7 (1.5) | 3.6 (2.1) | 2.6 (1.8) | 2.9 (2.0) |
| 28-Day mortality no. (%) | 4394 (14%) | 1191 (8%) | 2104 (23%) | 352 (8%) | 747 (25%) | 532 (5%) | 999 (13%) | 1579 (19%) | 1284 (33%) |
| 365-Day mortality no. (%) | 11655 (38%) | 3777 (27%) | 4714 (51%) | 1704 (38%) | 1460 (48%) | 2318 (22%) | 3203 (41%) | 3948 (46%) | 2186 (57%) |

© 2026 Yoon CH et al. *JAMA Network Open.*

**eTable 6.** Highlight Table of Pearson Correlation Coefficients Between Scaled Variable Values and Phenotype Assignment of the Oslo Cohort Using Phenotypes Derived From Each Site Where 26 Variables Were Available (Oslo, Oxford, Stockholm, SENECA). Correlation coefficients are shaded blue when positive, red when negative. Higher absolute values are shaded with greater intensities of their respective color. SBP: Systolic Blood Pressure, paO2: partial pressure of oxygen, SpO2: oxygen saturation, GCS: Glasgow Coma Scale, INR: International Normalized Ratio, CRP: C-Reactive Protein, ESR: Erythrocyte Sedimentation Rate, ALAT: Alanine Transaminases, ASAT: Aspartate Transaminases, BUN: Blood Urea Nitrogen, IU: International Units. Data source: Oslo, Variable set: 26v

| | **Oslo** | | | | **Oxford** | | | | **Stockholm** | | | | **SENECA** | | | |
|---|---|---|---|---|---|---|---|---|---|---|---|---|---|---|---|---|
| **variable** | **1os** | **2os** | **3os** | **4os** | **1ox** | **2ox** | **3ox** | **4ox** | **1st** | **2st** | **3st** | **4st** | **alpha** | **beta** | **gamma** | **delta** |
| Age | -0.12 | 0.24 | -0.11 | -0.06 | -0.23 | 0.22 | -0.17 | 0.10 | -0.19 | 0.22 | -0.20 | 0.11 | -0.20 | 0.25 | -0.04 | 0.02 |
| Sex, male | -0.09 | 0.02 | 0.10 | 0.02 | -0.07 | 0.03 | 0.01 | 0.05 | -0.02 | -0.01 | -0.03 | 0.07 | -0.04 | 0.04 | -0.05 | 0.08 |
| ALAT | -0.20 | -0.16 | 0.38 | 0.14 | -0.01 | -0.21 | 0.07 | 0.22 | -0.00 | -0.16 | 0.06 | 0.19 | -0.05 | -0.20 | -0.07 | 0.42 |
| Albumin | 0.47 | -0.40 | -0.27 | 0.11 | 0.51 | -0.36 | -0.17 | -0.09 | 0.55 | -0.40 | -0.15 | -0.09 | 0.56 | -0.11 | -0.34 | -0.20 |
| Bilirubin | -0.13 | -0.20 | 0.53 | -0.06 | 0.00 | -0.21 | 0.10 | 0.19 | 0.03 | -0.10 | 0.07 | 0.06 | -0.03 | -0.20 | -0.02 | 0.32 |
| BUN | -0.53 | 0.49 | 0.07 | 0.07 | -0.52 | 0.40 | -0.07 | 0.18 | -0.47 | 0.35 | -0.11 | 0.26 | -0.47 | 0.43 | -0.13 | 0.28 |
| Chloride | -0.03 | 0.06 | -0.12 | 0.08 | -0.01 | -0.12 | -0.10 | 0.20 | 0.03 | -0.18 | -0.07 | 0.28 | 0.04 | 0.10 | -0.23 | 0.11 |
| Creatinine | -0.45 | 0.46 | 0.03 | 0.02 | -0.41 | 0.40 | -0.05 | 0.04 | -0.38 | 0.31 | -0.06 | 0.13 | -0.38 | 0.45 | -0.17 | 0.18 |
| CRP | -0.20 | 0.29 | 0.13 | -0.27 | -0.40 | 0.30 | 0.08 | 0.08 | -0.37 | 0.41 | 0.02 | -0.08 | -0.46 | 0.07 | 0.41 | 0.02 |
| ESR | -0.14 | 0.39 | -0.07 | -0.28 | -0.43 | 0.45 | 0.09 | -0.08 | -0.40 | 0.51 | 0.03 | -0.18 | -0.46 | 0.28 | 0.36 | -0.19 |
| GCS | 0.24 | 0.12 | 0.04 | -0.65 | 0.04 | 0.18 | 0.04 | -0.29 | 0.09 | 0.20 | 0.04 | -0.46 | 0.00 | 0.12 | 0.13 | -0.33 |
| Glucose | -0.10 | -0.09 | -0.00 | 0.32 | -0.11 | -0.02 | 0.00 | 0.15 | -0.06 | -0.06 | -0.03 | 0.19 | -0.07 | -0.02 | -0.01 | 0.13 |
| Hemoglobin | 0.32 | -0.48 | -0.01 | 0.22 | 0.40 | -0.45 | -0.18 | 0.16 | 0.44 | -0.42 | -0.19 | 0.12 | 0.44 | -0.30 | -0.23 | 0.07 |
| HR | 0.07 | -0.23 | 0.16 | 0.06 | -0.01 | -0.27 | 0.11 | 0.28 | 0.11 | -0.20 | 0.05 | 0.11 | 0.01 | -0.34 | 0.25 | 0.10 |
| INR | -0.13 | -0.05 | 0.34 | -0.08 | -0.10 | -0.02 | 0.03 | 0.14 | -0.05 | 0.00 | 0.02 | 0.06 | -0.13 | -0.06 | 0.00 | 0.25 |
| Lactate | -0.33 | -0.09 | 0.32 | 0.34 | -0.21 | -0.17 | 0.04 | 0.44 | -0.18 | -0.16 | 0.03 | 0.49 | -0.16 | -0.21 | 0.01 | 0.48 |
| Leucocytes | 0.00 | 0.13 | -0.29 | 0.13 | -0.04 | 0.11 | -0.39 | 0.13 | 0.01 | 0.19 | -0.47 | 0.06 | -0.11 | -0.03 | 0.11 | 0.05 |
| paO2 | -0.21 | -0.03 | 0.01 | 0.40 | -0.00 | -0.02 | 0.03 | 0.01 | -0.11 | -0.07 | 0.06 | 0.23 | -0.04 | -0.03 | -0.13 | 0.26 |
| RR | 0.01 | -0.09 | 0.06 | 0.06 | -0.08 | -0.14 | -0.03 | 0.29 | 0.01 | -0.10 | -0.05 | 0.17 | -0.05 | -0.13 | 0.15 | 0.04 |
| SpO2 | 0.14 | -0.05 | -0.06 | -0.09 | -0.06 | -0.06 | -0.10 | 0.21 | 0.08 | -0.02 | -0.19 | 0.06 | 0.01 | -0.12 | 0.13 | -0.04 |
| SBP | 0.38 | -0.21 | -0.22 | -0.08 | 0.30 | -0.13 | -0.06 | -0.17 | 0.32 | -0.12 | -0.09 | -0.22 | 0.27 | 0.02 | -0.10 | -0.29 |
| Sodium | -0.01 | 0.01 | -0.13 | 0.15 | -0.01 | -0.17 | -0.16 | 0.31 | 0.04 | -0.21 | -0.13 | 0.37 | 0.05 | 0.06 | -0.23 | 0.16 |

© 2026 Yoon CH et al. *JAMA Network Open.*

| | Oslo | | | | Oxford | | | | Stockholm | | | | SENECA | | | |
|---|---|---|---|---|---|---|---|---|---|---|---|---|---|---|---|---|
| **variable** | **1os** | **2os** | **3os** | **4os** | **1ox** | **2ox** | **3ox** | **4ox** | **1st** | **2st** | **3st** | **4st** | **alpha** | **beta** | **gamma** | **delta** |
| Temperature | 0.28 | -0.12 | 0.02 | -0.32 | 0.09 | -0.13 | 0.09 | -0.00 | 0.20 | -0.07 | 0.08 | -0.25 | 0.11 | -0.10 | 0.15 | -0.23 |
| Thrombocytes | 0.05 | 0.19 | -0.41 | 0.09 | -0.00 | 0.17 | -0.50 | 0.06 | 0.03 | 0.21 | -0.49 | 0.03 | -0.04 | 0.05 | 0.10 | -0.15 |
| Troponin T | -0.21 | 0.13 | 0.02 | 0.14 | -0.17 | 0.04 | -0.06 | 0.19 | -0.17 | 0.00 | -0.04 | 0.29 | -0.17 | 0.01 | -0.08 | 0.34 |
| Bands | 0.05 | 0.08 | -0.17 | -0.03 | 0.02 | 0.10 | -0.38 | 0.07 | 0.05 | 0.11 | -0.33 | 0.01 | -0.07 | -0.05 | 0.09 | 0.04 |

© 2026 Yoon CH et al. *JAMA Network Open.*

**eTable 7.** Highlight Table of Pearson Correlation Coefficients Between Scaled Variable Values and Phenotype Assignment of the Oxford Cohort Using Phenotypes Derived From Sites Where 29 Variables Were Available (Oxford, Stockholm, SENECA). Correlation coefficients are shaded blue when positive, red when negative. Higher absolute values are shaded with greater intensities of their respective color. SBP: Systolic Blood Pressure, paO2: partial pressure of oxygen, SpO2: oxygen saturation, GCS: Glasgow Coma Scale, INR: International Normalized Ratio, CRP: C-Reactive Protein, ESR: Erythrocyte Sedimentation Rate, ALAT: Alanine Transaminases, ASAT: Aspartate Transaminases, BUN: Blood Urea Nitrogen, IU: International Units. Data source: Oxford, Variable set: 29v

| | **Oxford** | | | | **Stockholm** | | | | **SENECA** | | | |
|---|---|---|---|---|---|---|---|---|---|---|---|---|
| **variable** | **1ox** | **2ox** | **3ox** | **4ox** | **1st** | **2st** | **3st** | **4st** | **alpha** | **beta** | **gamma** | **delta** |
| Age | -0.04 | 0.21 | -0.17 | -0.09 | -0.03 | 0.20 | -0.21 | -0.06 | -0.23 | 0.21 | 0.03 | 0.01 |
| Sex, male | 0.00 | 0.03 | -0.03 | -0.02 | -0.02 | 0.04 | -0.03 | -0.01 | 0.01 | 0.02 | -0.04 | 0.02 |
| ALAT | -0.27 | -0.24 | 0.01 | 0.69 | -0.22 | -0.21 | 0.00 | 0.64 | -0.00 | -0.27 | -0.06 | 0.46 |
| Albumin | 0.34 | -0.29 | -0.00 | -0.08 | 0.31 | -0.28 | -0.01 | -0.06 | 0.42 | -0.11 | -0.28 | -0.07 |
| Bilirubin | -0.09 | -0.32 | -0.02 | 0.57 | -0.06 | -0.30 | -0.02 | 0.55 | 0.08 | -0.30 | -0.00 | 0.29 |
| BUN | -0.44 | 0.62 | -0.16 | -0.11 | -0.43 | 0.64 | -0.21 | -0.07 | -0.55 | 0.50 | -0.02 | 0.15 |
| Chloride | -0.08 | 0.17 | -0.07 | -0.07 | -0.17 | 0.26 | -0.08 | -0.03 | -0.04 | 0.03 | -0.14 | 0.21 |
| Creatinine | -0.39 | 0.57 | -0.16 | -0.11 | -0.42 | 0.60 | -0.18 | -0.06 | -0.44 | 0.52 | -0.13 | 0.10 |
| CRP | -0.34 | 0.31 | 0.03 | 0.04 | -0.13 | 0.19 | -0.06 | -0.03 | -0.43 | 0.02 | 0.39 | 0.05 |
| ESR | -0.44 | 0.43 | 0.12 | -0.06 | -0.26 | 0.30 | 0.08 | -0.11 | -0.51 | 0.26 | 0.36 | -0.10 |
| GCS | -0.13 | 0.02 | 0.12 | 0.05 | -0.05 | -0.04 | 0.12 | 0.03 | -0.05 | 0.11 | 0.07 | -0.16 |
| Glucose | -0.23 | 0.31 | -0.13 | -0.01 | -0.17 | 0.28 | -0.16 | 0.00 | -0.26 | 0.10 | 0.07 | 0.14 |
| Hemoglobin | 0.48 | -0.34 | -0.33 | 0.05 | 0.40 | -0.27 | -0.30 | 0.06 | 0.41 | -0.21 | -0.24 | 0.02 |
| Heart rate | -0.13 | 0.00 | 0.06 | 0.12 | -0.03 | -0.03 | 0.01 | 0.08 | -0.09 | -0.31 | 0.27 | 0.18 |
| INR | -0.16 | 0.11 | -0.06 | 0.11 | -0.15 | 0.10 | -0.07 | 0.15 | -0.18 | 0.00 | 0.03 | 0.22 |
| Lactate | -0.17 | 0.04 | -0.13 | 0.28 | -0.22 | 0.12 | -0.14 | 0.31 | -0.17 | -0.21 | 0.03 | 0.49 |
| Leucocytes | -0.06 | 0.18 | -0.34 | 0.09 | 0.08 | 0.16 | -0.46 | 0.08 | -0.18 | -0.04 | 0.12 | 0.15 |
| paO2 | -0.01 | 0.03 | -0.01 | -0.01 | -0.11 | 0.07 | 0.05 | 0.02 | -0.02 | 0.02 | -0.07 | 0.10 |
| RR | -0.07 | 0.09 | -0.08 | 0.04 | -0.01 | 0.08 | -0.13 | 0.02 | -0.18 | -0.19 | 0.22 | 0.21 |
| SpO2 | -0.06 | 0.10 | -0.05 | -0.01 | 0.11 | 0.01 | -0.16 | -0.04 | -0.12 | -0.11 | 0.22 | 0.02 |
| SBP | 0.25 | -0.17 | -0.07 | -0.06 | 0.18 | -0.14 | -0.04 | -0.05 | 0.25 | 0.13 | -0.29 | -0.15 |
| Sodium | 0.00 | 0.10 | -0.06 | -0.10 | -0.05 | 0.17 | -0.09 | -0.08 | 0.01 | 0.02 | -0.11 | 0.12 |
| Temperature | -0.01 | -0.07 | 0.15 | -0.00 | 0.15 | -0.18 | 0.09 | -0.05 | 0.05 | -0.22 | 0.23 | -0.09 |
| Thrombocytes | 0.15 | 0.20 | -0.63 | -0.01 | 0.19 | 0.19 | -0.60 | -0.01 | -0.07 | 0.13 | -0.03 | -0.02 |
| Troponin T | -0.09 | 0.06 | -0.06 | 0.08 | -0.13 | 0.09 | -0.07 | 0.14 | -0.11 | -0.05 | -0.07 | 0.32 |
| Bands | 0.09 | 0.16 | -0.56 | 0.08 | 0.12 | 0.12 | -0.46 | 0.06 | -0.13 | -0.01 | 0.09 | 0.08 |
| ASAT | -0.28 | -0.25 | -0.05 | 0.74 | -0.26 | -0.19 | -0.04 | 0.72 | -0.03 | -0.26 | -0.10 | 0.53 |
| Bicarbonate | 0.33 | -0.35 | 0.10 | -0.06 | 0.39 | -0.42 | 0.11 | -0.10 | 0.30 | -0.15 | 0.09 | -0.34 |
| Elixhauser | -0.26 | 0.31 | -0.09 | -0.01 | -0.27 | 0.33 | -0.08 | 0.01 | -0.29 | 0.30 | -0.02 | 0.05 |

© 2026 Yoon CH et al. *JAMA Network Open.*

**eTable 8.** Highlight Table of Pearson Correlation Coefficients Between Scaled Variable Values and Phenotype Assignment of the Stockholm Cohort Using Phenotypes Derived From Sites Where 29 Variables Were Available (Oxford, Stockholm, SENECA). Correlation coefficients are shaded blue when positive, red when negative. Higher absolute values are shaded with greater intensities of their respective color. SBP: Systolic Blood Pressure, paO2: partial pressure of oxygen, SpO2: oxygen saturation, GCS: Glasgow Coma Scale, INR: International Normalized Ratio, CRP: C-Reactive Protein, ESR: Erythrocyte Sedimentation Rate, ALAT: Alanine Transaminases, ASAT: Aspartate Transaminases, BUN: Blood Urea Nitrogen, IU: International Units. Data source: Stockholm, Variable set: 29v

| | Oxford | | | | Stockholm | | | | SENECA | | | |
|---|---|---|---|---|---|---|---|---|---|---|---|---|
| variable | 1ox | 2ox | 3ox | 4ox | 1st | 2st | 3st | 4st | alpha | beta | gamma | delta |
| Age | -0.10 | 0.30 | -0.23 | -0.05 | -0.08 | 0.30 | -0.26 | -0.01 | -0.31 | 0.23 | 0.03 | 0.10 |
| Sex, male | -0.01 | 0.02 | -0.02 | 0.01 | -0.04 | 0.05 | -0.02 | 0.02 | 0.02 | 0.04 | -0.08 | 0.03 |
| ALAT | -0.18 | -0.28 | 0.04 | 0.64 | -0.18 | -0.24 | 0.06 | 0.61 | 0.04 | -0.25 | -0.14 | 0.45 |
| Albumin | 0.43 | -0.37 | 0.01 | -0.12 | 0.34 | -0.32 | 0.02 | -0.09 | 0.54 | -0.09 | -0.37 | -0.15 |
| Bilirubin | -0.12 | -0.24 | -0.04 | 0.57 | -0.14 | -0.21 | -0.04 | 0.59 | -0.00 | -0.20 | -0.08 | 0.38 |
| BUN | -0.45 | 0.64 | -0.25 | -0.01 | -0.44 | 0.66 | -0.27 | 0.03 | -0.56 | 0.51 | -0.07 | 0.22 |
| Chloride | -0.06 | 0.18 | -0.10 | -0.06 | -0.16 | 0.27 | -0.10 | -0.01 | -0.04 | 0.07 | -0.19 | 0.21 |
| Creatinine | -0.34 | 0.52 | -0.22 | -0.03 | -0.37 | 0.57 | -0.23 | 0.01 | -0.38 | 0.47 | -0.18 | 0.17 |
| CRP | -0.27 | 0.30 | -0.05 | 0.01 | -0.09 | 0.20 | -0.08 | -0.06 | -0.43 | 0.10 | 0.37 | -0.01 |
| ESR | -0.29 | 0.32 | 0.10 | -0.13 | -0.10 | 0.18 | 0.08 | -0.20 | -0.41 | 0.23 | 0.39 | -0.23 |
| GCS | -0.01 | -0.05 | 0.10 | -0.01 | 0.07 | -0.13 | 0.11 | -0.04 | 0.05 | 0.07 | 0.07 | -0.26 |
| Glucose | -0.11 | 0.21 | -0.15 | 0.01 | -0.07 | 0.19 | -0.16 | 0.02 | -0.17 | 0.04 | 0.04 | 0.13 |
| Hemoglobin | 0.45 | -0.20 | -0.41 | 0.05 | 0.41 | -0.16 | -0.41 | 0.05 | 0.29 | -0.15 | -0.18 | 0.03 |
| Heart rate | -0.04 | -0.01 | -0.00 | 0.07 | 0.06 | -0.06 | -0.03 | 0.03 | -0.06 | -0.34 | 0.31 | 0.11 |
| INR | -0.13 | 0.06 | -0.09 | 0.19 | -0.12 | 0.06 | -0.10 | 0.24 | -0.15 | -0.02 | -0.02 | 0.27 |
| Lactate | -0.20 | 0.10 | -0.15 | 0.31 | -0.23 | 0.16 | -0.17 | 0.33 | -0.21 | -0.20 | 0.03 | 0.51 |
| Leucocytes | 0.15 | 0.26 | -0.67 | 0.09 | 0.25 | 0.24 | -0.73 | 0.08 | -0.21 | 0.07 | 0.07 | 0.12 |
| paO2 | -0.14 | 0.04 | 0.09 | 0.05 | -0.25 | 0.12 | 0.12 | 0.10 | -0.06 | 0.07 | -0.14 | 0.19 |
| RR | 0.00 | 0.11 | -0.20 | 0.04 | 0.09 | 0.08 | -0.24 | 0.03 | -0.16 | -0.21 | 0.24 | 0.18 |
| SpO2 | 0.14 | 0.10 | -0.31 | -0.04 | 0.26 | 0.03 | -0.37 | -0.05 | -0.09 | -0.14 | 0.21 | 0.04 |
| SBP | 0.19 | -0.16 | 0.01 | -0.06 | 0.18 | -0.17 | 0.02 | -0.06 | 0.19 | 0.13 | -0.20 | -0.19 |
| Sodium | 0.04 | 0.11 | -0.11 | -0.10 | -0.05 | 0.18 | -0.12 | -0.06 | 0.03 | 0.06 | -0.18 | 0.14 |
| Temperature | 0.15 | -0.21 | 0.13 | -0.04 | 0.22 | -0.27 | 0.12 | -0.09 | 0.20 | -0.22 | 0.13 | -0.18 |
| Thrombocytes | 0.21 | 0.27 | -0.73 | 0.04 | 0.30 | 0.23 | -0.73 | 0.02 | -0.20 | 0.08 | 0.10 | 0.04 |
| Troponin T | -0.14 | 0.11 | -0.09 | 0.14 | -0.16 | 0.12 | -0.10 | 0.19 | -0.16 | 0.01 | -0.06 | 0.30 |
| Bands | 0.16 | 0.18 | -0.58 | 0.09 | 0.18 | 0.15 | -0.51 | 0.08 | -0.12 | -0.03 | 0.09 | 0.09 |
| ASAT | -0.20 | -0.17 | -0.17 | 0.71 | -0.18 | -0.13 | -0.17 | 0.70 | -0.12 | -0.21 | -0.10 | 0.58 |
| Bicarbonate | 0.28 | -0.26 | 0.11 | -0.14 | 0.34 | -0.34 | 0.11 | -0.19 | 0.23 | -0.08 | 0.12 | -0.38 |
| Elixhauser | -0.29 | 0.36 | -0.09 | 0.00 | -0.28 | 0.37 | -0.10 | 0.02 | -0.32 | 0.31 | -0.01 | 0.06 |

© 2026 Yoon CH et al. *JAMA Network Open.*

**eReference.**

1. Seymour CW, Kennedy JN, Wang S, et al. Derivation, Validation, and Potential Treatment Implications of Novel Clinical Phenotypes for Sepsis. JAMA 2019;**321**(20):2003–17 doi: 10.1001/jama.2019.5791.

© 2026 Yoon CH et al. *JAMA Network Open.*
