# Supplementary material for: Multicenter Validation of Clinical Sepsis Phenotypes
Source: JAMA Netw Open. 2026 Jun 1;9(6):e2616134. doi: 10.1001/jamanetworkopen.2026.16134 (PMC13227316; doi:10.1001/jamanetworkopen.2026.16134)
Supplement: Supplement 2. — Data Sharing Statement [file jamanetwopen-e2616134-s002.pdf]

Data Sharing Statement

Yoon. Multicenter Validation of Clinical Sepsis Phenotypes. *JAMA Netw Open*. Published June 01, 2026. doi:10.1001/jamanetworkopen.2026.16134

Data
Data available: No
